# Characterization of *DvSSJ1* transcripts targeting the smooth septate junction (SSJ) of western corn rootworm (*Diabrotica virgifera virgifera*)

**DOI:** 10.1038/s41598-020-68014-1

**Published:** 2020-07-07

**Authors:** Xu Hu, Chad J. Boeckman, Bin Cong, Joe P. Steimel, Nina M. Richtman, Kristine Sturtz, Yiwei Wang, Carl A. Walker, Jiaming Yin, Anita Unger, Caitlin Farris, Albert L. Lu

**Affiliations:** Corteva Agriscience, 7300 NW 62nd Ave., Johnston, IA 50131 USA

**Keywords:** Biotechnology, Plant sciences

## Abstract

Transgenic maize plants expressing dsRNA targeting western corn rootworm (WCR, *Diabrotica virgifera virgifera*) *DvSSJ1* mRNA*,* a *Drosophila* snakeskin (*ssk*) ortholog, show insecticidal activity and significant plant protection from WCR damage. The gene encodes a membrane protein associated with the smooth sepate junction (SSJ) which is required for intestinal barrier function. To understand the active RNA form that leads to the mortality of WCR larvae by *DvSSJ1* RNA interference (RNAi), we characterized transgenic plants expressing *DvSSJ1* RNA transcripts targeting WCR *DvSSJ1* mRNA. The expression of the silencing cassette results in the full-length transcript of 901 nucleotides containing a 210 bp inverted fragment of the *DvSSJ1* gene, the formation of a double-stranded RNA (dsRNA) transcript and siRNAs in transgenic plants. Our artificial diet-feeding study indicates that dsRNAs greater than or equal to approximately 60 base-pairs (bp) are required for *DvSSJ1* insecticidal activity. Impact of specificity of dsRNA targeting *DvSSJ1* mRNA on insecticidal activities was also evaluated in diet bioassay, which showed a single nucleotide mutation can have a significant impact or abolish diet activities against WCR. These results provide insights as to the functional forms of plant-delivered dsRNA for the protection of transgenic maize from WCR feeding damage and information contributing to the risk assessment of transgenic maize expressing insecticidal dsRNA.

## Introduction

RNA interference (RNAi) is a sequence-specific gene silencing mechanism in eukaryotes that is triggered by double-stranded RNA (dsRNA)^1^ and has become an important tool for reverse functional genomics and applications in biomedicine and agriculture^2,3^. The dsRNA is first processed by the nuclease Dicer in an ATP-dependent reaction that cleaves long dsRNA into small interfering RNA (siRNA) fragments of 21–26 nucleotides (nt) in length with a 2 nt overhang on the 3′ end. These siRNA fragments are further processed by the RNA-induced silencing complex (RISC) into two single-stranded RNAs, the passenger strand and the guide strand. The guide strand binds with an Argonaute (AGO) protein, the catalytic component of RISC, while the passenger strand is degraded by AGO. The guide strand complex is then paired with its homologous messenger RNA sequence by precise complementation resulting in the endonucleolytic cleavage of the target mRNA^4^.

The western corn rootworm (WCR), *Diabrotica virgifera virgifera* (Coleoptera: Chrysomelidae), is one of the most economically important and invasive pests of maize throughout the US Corn Belt^5^. WCR shows a robust systemic RNAi response induced by direct injection of dsRNA^6^, oral feeding of dsRNA provided in artificial diet^7,8^, or transgenic plants expressing dsRNA^7,8^. By its very nature, RNAi insect control technology represents a different mode of action than protein-based traits (*i.e.* Cry proteins from *Bacillus thuringiensis*). The putative *Drosophila* snakeskin (*ssk*) homolog of WCR (*DvSSJ1*) has previously been shown to be an effective RNAi target for corn rootworm control^8,9^. The target gene, *DvSSJ1*, encodes a membrane protein associated with the smooth sepate junction (SSJ) which is required for intestinal barrier function. Three SSJ-associated membrane proteins, Ssk, Mesh, and Tsp2A, have been reported in *Drosophila*^10^ and their homologous counterparts were identified from WCR^9^. These proteins play a crucial role in maintaining tissue homeostasis through the regulation of stem cell proliferation and enterocyte behavior in the *Drosophila* adult midgut^11^. When the western corn rootworm feeds on *DvSSJ1* dsRNA, the smooth septate junction gene is down-regulated by the insect’s RNAi machinery resulting in pest mortality^9^.

Transgenic plants expressing dsRNA targeting *DvSSJ1* show insecticidal activity and significant plant protection from WCR damage^8^. Diet based insect feeding assays using dsRNA targeting *DvSSJ1* mRNA demonstrate that SSJ protein accumulation and mortality was negatively correlated and that the interactions of SSJ proteins and barrier function are impaired in RNAi-compromised or knockout insects^9^. These findings support that the malfunction of SSJ complexes in the midgut triggered by *DvSSJ1* RNAi were the main effects leading to the death of WCR. The *DvSSJ1* is a midgut-specific gene in WCR and its functions are consistent with the biological functions described for *Drosophila*^9^. Two forms of transgenic *DvSSJ1* dsRNAs present in plant tissues are long dsRNAs and siRNAs^8^. Similar expression patterns have been reported for *Snf7*^7^ and *v-ATPase C*^12^ in transgenic maize plants*.* Diet-based studies of *Snf7*^13^ and *v-ATPase C*^12^ showed that dsRNA of at least 60 bp in length resulted in high levels of larval mortality, while dsRNAs with lengths shorter than 60 bp did not cause a significant RNAi response. Although the mechanism of uptake at the cellular level is still unknown, an inefficient RNAi response with short dsRNA or 21-bp siRNA demonstrated less uptake in larval midgut cells for both snf7^13^ and *DvSSJ1*^9^.

The goal of the current study was to understand the active RNA form of transgenic plants expressing *DvSSJ1* targeting the SSJ of WCR by analyzing: (1) sequences and expression of *DvSSJ1* transcripts in transgenic plants; (2) functional forms of RNAs targeting the SSJ of WCR *in planta*; and (3) impacts of length of dsRNA and specificity (mismatches) of 21 bp siRNAs embedded in the Green Fluorescence Protein (GFP) sequence on insecticidal activity in artificial diet. Our data indicate that a single mismatch in siRNA (21mer) embedded in GFP sequence can significantly reduce insecticidal activity and dsRNAs greater than or equal to approximately 60 base-pairs (bp) are required for larval mortality in the artificial diet bioassays. Furthermore, transgenic plants only expressing certain 21 bp siRNAs matching the target sequence (*DvSSJ1*) were not efficacious. These results provide additional implications for the effectiveness of dsRNA for transgenic maize to control WCR feeding damage and contribute to the risk assessment of transgenic maize expressing insecticidal dsRNA.

## Results and discussion

Molecular characterization of the transgenic plants expressing *DvSSJ1* and understanding the active form of *DvSSJ1* dsRNA against WCR *in planta* is important for the risk assessment of transgenic maize expressing insecticidal dsRNA and the effective design of RNAi hairpin constructs for WCR control. To gain insight into the functional forms of dsRNA effective against WCR artificial diet-based feeding and transgenic plant-based testing were performed to identify the active forms of *DvSSJ1* dsRNA against WCR. Northern blot and sequence analyses of *DvSSJ1* dsRNA derived from transgenic maize indicated the presence of detectable levels of both intact dsRNA and plant-processed siRNAs. This posed the question of which RNA form *in planta*, full-length dsRNA or siRNA, or both, caused the RNAi response in WCR.

### Characterization of *DvSSJ1* dsRNA expression in transgenic maize

**Figure 1:**
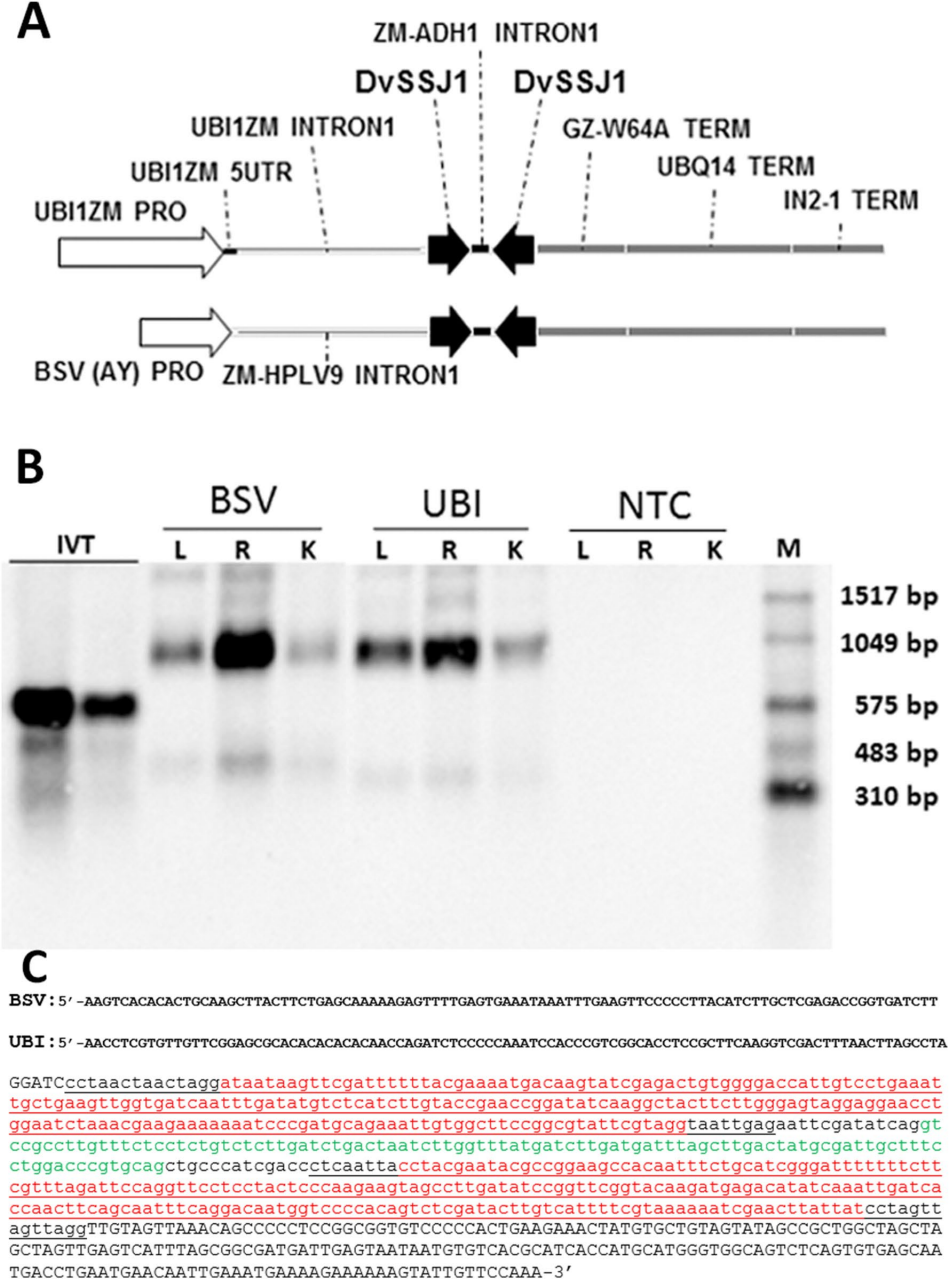
Charactering expression of *DvSSJ1* transcript in T1 transgenic plants. (**A)** Diagram of *DvSSJ1* expression cassette containing a promoter and terminators; Intron region from the *Zea mays* ortholog of an *Oryza sativa* (rice) hypothetical protein (ZM-HPLV9); (**B**) Northern analysis of DvSSJ1 expression in transgenic tissues collected from leaf (L), root (R), and kernel (K); purified mRNAs (200 ng) were compared to in vitro transcription (IVT; 10 pg and 5 pg) of *DvSSJ1* control and RNA size marker (M). Plants of non-transgenic control (NTC) were included. The blot was probed with the 210 bp *DvSSJ1* antisense riboprobe labeled with digoxigenin. The estimated amount (pg) of the top band were analyzed by ImageJ. (**C**) The sequence of the full *DvSSJ1* transcript was analyzed by both cDNA sequencing and 5′ and 3′ RACE (Supplementary Fig. 2). The 901-nucleotide (nt) of full *DvSSJ1* transcript (UBI promoter; 900 nt for BSV) contains the 5′ or 3′ end of additional sequences shown in black and capital letter, which are partially matched to 5UTR of maize polyubiquitin (**C**; S94464.1) and terminator of maize zein Zc2 gene (**D**; X53514.1) as part of the construct elements. Underline part of the sequence (232 bp) can form dsRNA stem and loop region in the middle (590 nt). *DvSSJ1* 210 bp was highlighted in red and truncated maize ADH1 intron1 in green.

**Figure 2:**
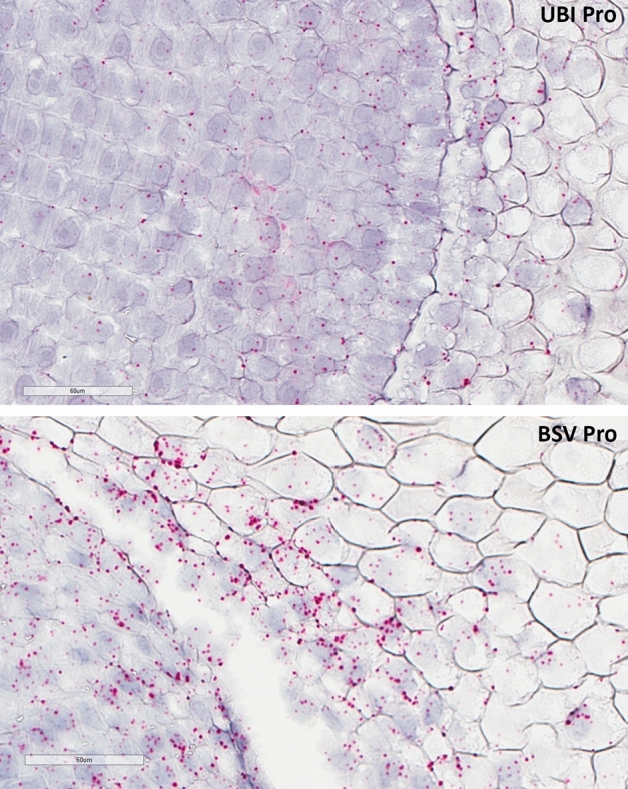
Visualization of *DvSSJ1* transcript in the transgenic root tips. Transgenic root samples were collected at the V6 stage in the greenhouse and hybridized with *DvSSJ1* probe. Single red dot presents one *DvSSJ1* transcript in the root cell. Scale bar = 60 µm.

We previously reported that transgenic plants expressing dsRNA targeting *DvSSJ1* show insecticidal activity and significant plant protection from WCR damage^8^. In this study, the *DvSSJ1* expression cassette (Fig. 1A) contained the *DvSSJ1* inverted stem-loop configuration under control of either a ZmUBI or BSV(AY)^14^ promoter with a triple terminator set as described in the Methods. These terminators were used to reduce any impact on transcriptional interference^15^ of a downstream expression cassette. Single copy T0 transformants showing efficacy against WCR^8^ were selected for further characterization at the T1 generation. To understand the active form of the *DvSSJ1* RNA molecule triggering WCR mortality, we characterized the full-length *DvSSJ1* transcript produced in T1 transgenic plants using 5′and 3′ rapid amplification of cDNA ends (RACE) (Supplementary Fig. 1 and 2A) and subsequent cDNA sequencing. The results from the sequencing of cDNA representing the long dsRNA transcripts revealed additional 5′sequence upstream of the expected *DvSSJ1* dsRNA region (Fig. 1C). The presence of this additional sequence was confirmed by treatment of the transcript with RNase I_f_ which resulted in the removal of the single-strand region and produced a lower migrating band in Northern blots (Supplementary Fig. 1B). The molar ratio of the full-length transcript (top band = 901nt [UBI] and 900nt [BSV]) to the dsRNA hairpin ( bottom band = 590nt) was variable and likely influenced by different RNA isolation and probe preparation methods (Fig. 1B). The 901-nucleotides (nt) of the ZmUBI::*DvSSJ1* transcript contains additional nucleotide sequences at the 5′ and 3′ ends, shown in black and capital letters (Fig. 1C), that match to a portion of the 5′ UTR of the maize polyubiquitin (S94464.1) gene and to the maize zein Zc2 gene (X53514.1) terminator both of which are elements found in the expression construct (Supplementary Fig. 2B and 2C). As expected, the only differences identified between ZmUBI and BSV promoter-driven constructs were at the 5′ end of the transcript (Fig. 1C). A probe targeting the 3′ end of the *DvSSJ1* transcript (Supplementary Fig. 1A) was used to visualize *DvSSJ1* expression in the transgenic root tips using in situ hybridization (Fig. 2 and Supplementary Fig. 3–4). The results showed the strongest expression/accumulation of *DvSSJ1* transcript in the root apical meristem region (Supplementary Fig. 4A) while the mature root region showed weaker expression mostly near vascular bundle cells (Supplementary Fig. 4B).

**Figure 3:**
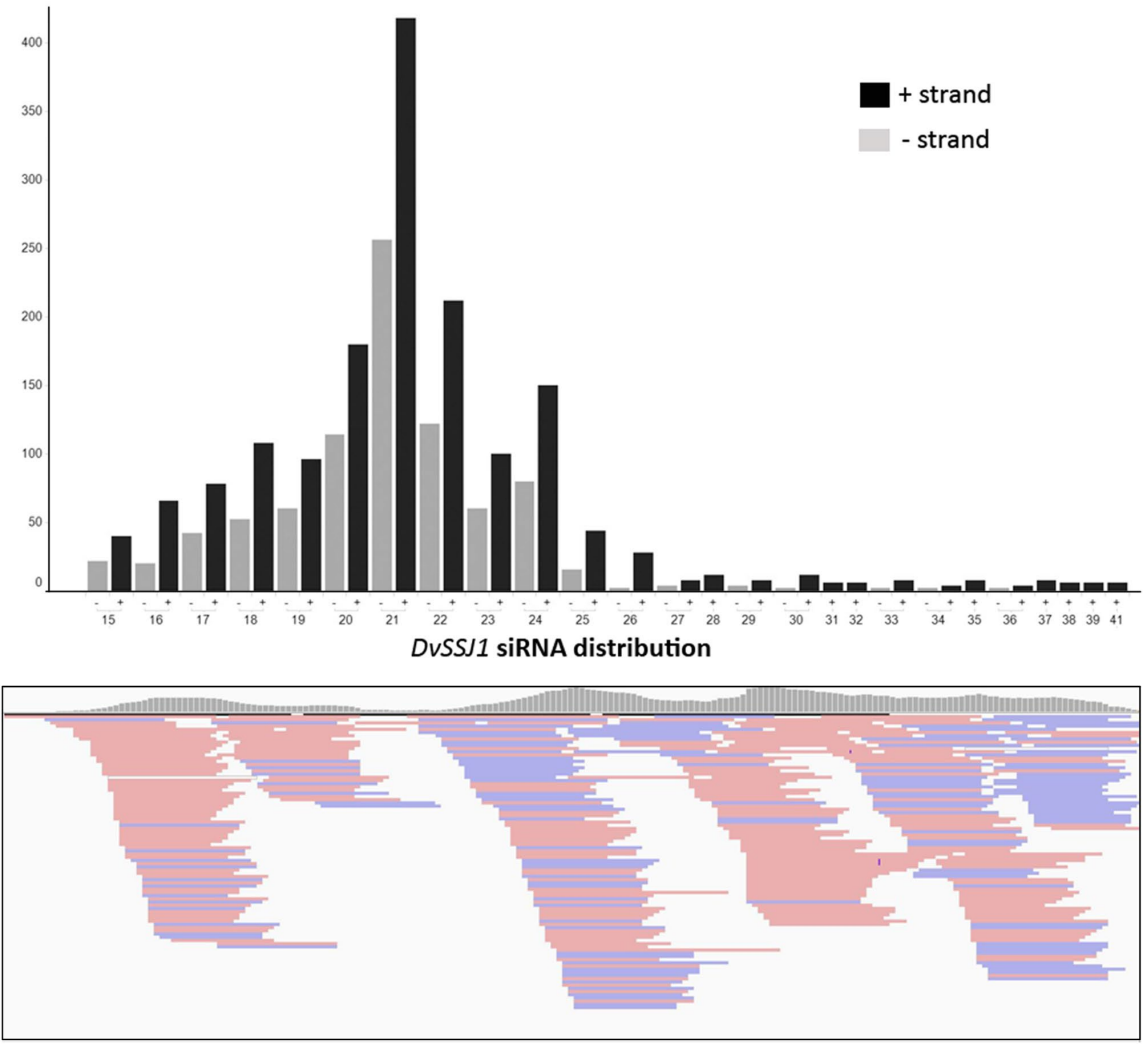
Analyses of *DvSSJ1* siRNA expression in transgenic plants. Size distribution and strand orientation of *DvSSJ1* small RNAs between 15 and 41 nucleotides were collected from root tissue under the control of the UBI promoter and showed in the top panel. Sense (red) and antisense (blue) of *DvSSJ1* siRNAs were mapped to 210 bp *DvSSJ1* fragment (Bottom panel). *DvSSJ1* siRNA reads were visualized using Integrative Genomics Viewer software 2.8 (Broad Institute, Cambridge, MA, USA) (https://software.broadinstitute.org/software/igv/).

**Figure 4:**
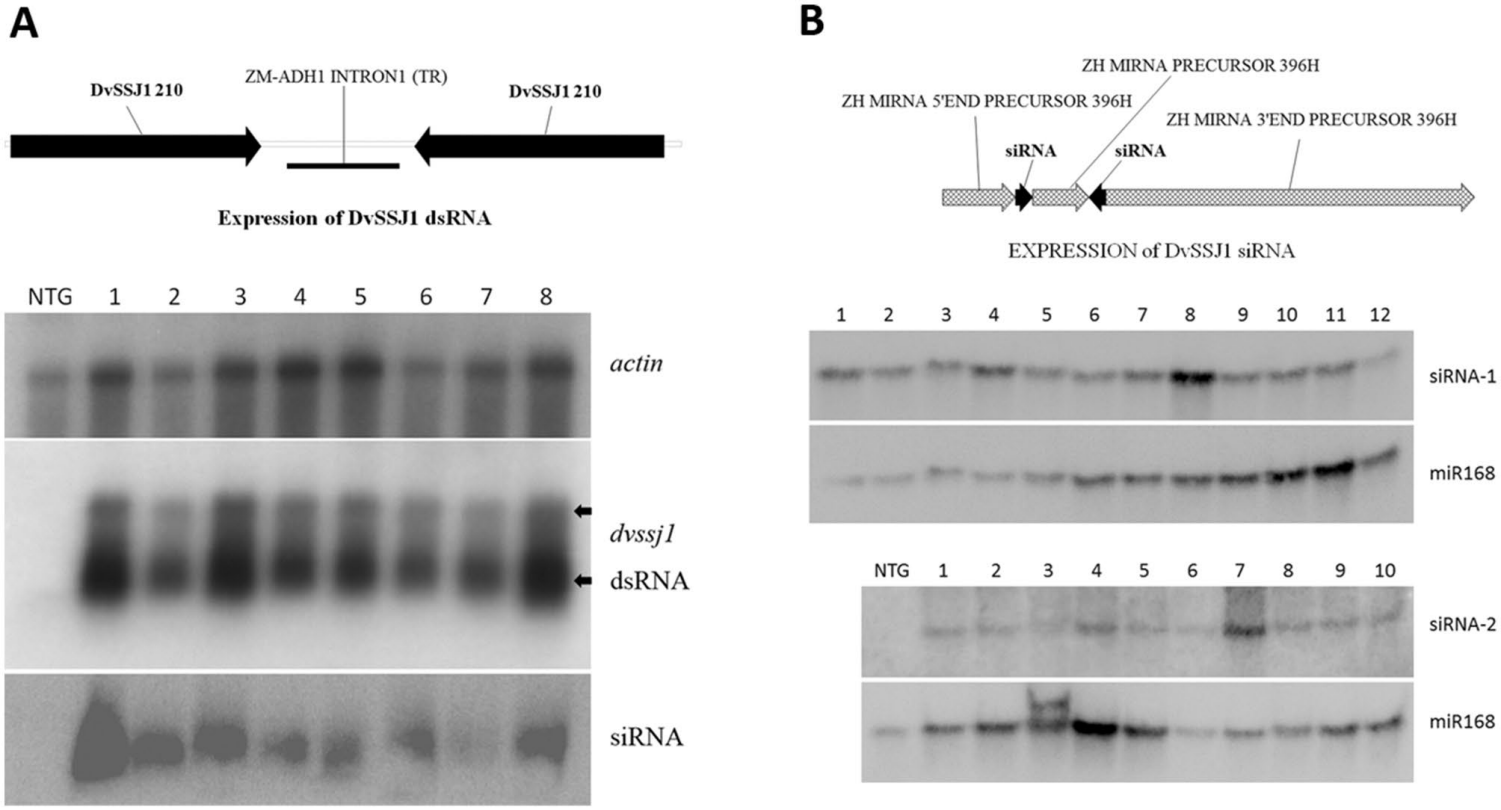
Northern blot analyses of T0 *DvSSJ1* root samples. (**A**) Northern blots of eight *DvSSJ1* dsRNA events and a non-transgenic control (NTC). A top diagram illustrates the dsRNA expression cassette. *Zm-actin* (Accession #: EU952376) was included as a reference gene for northern analysis (top panel). Plants containing 210 bp *DvSSJ1* cassette expressed both long dsRNA transcripts (middle panel) and siRNAs (bottom panel). There were two long dsRNA bands representing full *DvSSJ1* transcript (top arrow) and dsRNA hairpin (bottom arrow). (**B**) Northern blots of *DvSSJ1* siRNA containing events and a non-transgenic control (NTC). Two separate artificial miRNA constructs were designed to express 21-bp *DvSSJ1* siRNA-1 (TCCTTGATATCCGGTTCGGTA) and siRNA-2 (TAGTAGCCTTGATATCCGGTT). Exiqon LNA 5′ Biotin-labelled DNA probes (5 ng ml^−1^) were used for siRNA northern analyses and zm-miR168 was included as an internal control (Supplementary Table 3).

*DvSSJ1* siRNAs in plants were detected by small RNA sequencing (Fig. 3) and siRNA northern analysis (Fig. 4B). The 18–41-nt siRNA sequences mapped to the sense and antisense strands of the *DvSSJ1* fragment sequence (Fig. 3). The prevalent species of siRNA appeared to be 21-nt fragments, consistent with previous findings in transgenic maize plants containing RNAi constructs^7,12^. These siRNAs were generally distributed across the 210 bp-length of the *DvSSJ1* fragment sequence but there were specific sequence regions accumulating higher siRNA species than others. There were no significant changes in patterns of *DvSSJ1* siRNA accumulation between the different evaluated promoters or terminators (Supplementary Fig. 5 and 6B), and leaf or root tissue types (Supplementary Fig. 6A). The contribution of the promoter, hairpin dsRNA sequences and/or relative dynamics of dsRNA formation, Dicer processing, and degradation may impact the level of intact full-length *DvSSJ1* dsRNA and siRNAs in transgenic maize plants.

### Comparison of homologous sequences of *DvSSJ1* from other insect groups

A bioinformatics approach was used to assess how conserved the SSJ gene was across different organisms with varied evolutionary distance from western corn rootworm. We identified and selected homologs of *DvSSJ1* from published data^8,16^ and carried out transcriptome sequencing for six additional insects (Supplementary Fig. 7) representing four different orders (Supplementary Table 1). The sequences of twenty species, representing four families within the order *Coleoptera*, four families within the order *Lepidoptera*, and one family each from the order *Hymenoptera* and *Hemiptera* were compared to the 210 bp sequence from the WCR SSJ gene to determine the percent similarity, number of single nucleotide polymorphisms (SNPs), and the number of 21nt matches. In silico analysis was focused primarily within the order Coleoptera, with a total of 13 species assessed, because the *DvSSJ1* dsRNA is targeted to match the sequence of the smooth septate junction protein 1 (*DvSSJ1*) gene from WCR. The closest genomic sequence match (percent identity to the 210 bp *DvSSJ1* dsRNA sequence) was WCR (*D. virgifera virgifera)*, which as expected had a 100% sequence match, 0 SNPs, and 190 21nt matches to the *DvSSJ1* dsRNA sequence (Table 1). The *ssj1* gene from the closely related species, northern corn rootworm (NCR; *D. barberi*), shared 97.1% identity with the *DvSSJ1* dsRNA sequence, with 6 SNPs and 135 21nt matches. The *ssj1* gene from the southern corn rootworm (SCR; *D. undecimpunctata*) shared 92.9% identity with the *DvSSJ1* dsRNA sequence, with 15 SNPs and 79 21nt matches. The s*sj* genes from the other Coleoptera within the family Chrysomelidae [Colorado potato beetle (*Leptinotarsa decemlineata*), Crucifer flea beetle (*Phyllotreta cruciferae*), and Striped flea beetle (*Phyllotreta striolata*)] as well as species within the family Tenebrionidae, the family Coccinellidae, and the family Staphylinidae had decreasing percent identity with the *DvSSJ1* dsRNA sequence, ranging from 77.6 to 61.9% similarity and an increasing number of SNPs (ranging from 47 to 80). All Lepidoptera species within the four familes, as well as the honey bee (*Apis mellifera)* and the Insidious flower bug (*Orius insidiosus)* also had lower percent identity with the *DvSSJ1* dsRNA sequence, ranging from 64.8 to 60% identity, and an increased number of SNPs (ranging from 74 to 84). There were zero 21nt matches observed across all of the non-*Diabrotica* species analyzed.

**Table 1:** Sequence comparison of *DvSSJ1* homologous.

Order	Name	Percent identity to *DvSSJ1* dsRNA	Number of SNPs	Number of 21 nt matches (or longest nt sequence)*
Coleoptera	*Diabrotica virgifera virgifera*	100	0	190
	*Diabrotica longicornis*	97.1	6	135
	*Diabrotica undecimpunctata*	92.9	15	79
	*Phyllotreta cruciferae*	77.6	47	0 (20)
	*Phyllotreta striolata*	76.2	50	0 (19)
	*Leptinotarsa decemlineata*	73.3	56	0 (12)
	*Tribolium castaneum*	69.5	64	0 (11)
	*Zophobas morio*	69	65	0 (10)
	*Epilachna varivestis*	67.6	68	0 (12)
	*Tenebrio molitor*	65.2	73	0 (10)
	*Dalotia coriaria*	64.3	75	0 (8)
	*Cryptolaemus montrouzieri*	63.3	77	0 (8)
	*Coleomegilla maculata*	61.9	80	0 (13)
Hymenoptera	*Apis mellifera*	68.1	67	0 (10)
Hemiptera	*Orius insidious*	61.9	80	0 (11)
Lepidoptera	*Vanessa cardui*	64.8	74	0 (8)
	*Ostrinia nubilalis*	64.2	79	0 (9)
	*Spodoptera fugiperda*	62.9	78	0 (11)
	*Cydia pomonella*	60.5	83	0 (8)
	*Helicoverpa zea*	60	84	0 (8)

The sequences of twenty species, representing four families within the order *Coleoptera*, four families within the order *Lepidoptera*, and one family each from the order *Hymenoptera* and *Hemiptera* were compared to the 210 bp sequence from the WCR *SSJ* gene to determine the percent similarity, number of single nucleotide polymorphisms (SNPs), and the number of 21nt matches.

*Longest nucleotide sequence showing 100% match to *DvSSJ1* 210 sequence.

### Toxicity of *DvSSJ1* dsRNA against the target organism

#### dsRNA length requirements

To assess the relationship of dsRNA fragment length and insecticidal activity, a series of seven different lengths of *DvSSJ1* dsRNA was generated as described in the Method and Supplementary Fig. 9A and 10. WCR neonates were provided with seven different lengths of *DvSSJ1* dsRNA incorporated into a standard artificial diet as described in Method. Acceptable control mortality of WCR associated with this diet was set at 30% given the variability of WCR performance in laboratory bioassays^17^. WCR mortality was significantly greater than the control for each test substance at 60 base pairs (bp) of length and greater (Table 2). There was a statistically significant decrease in weight of WCR fed the *DvSSJ1* 40 bp fragment (*P* = 0.0448) though mortality was not affected (*P* = 0.3457). While the mortality associated with the 21 bp treatment exceeded the 30% acceptability criteria, this was not significant (*P* = 0.1988). A minimum length of dsRNA for efficacious RNAi activity in WCR diet was estimated at about 60-bp. This result is similar to that reported for *Snf7*^13^ and *Dv v-ATPase C*^12^ in WCR. This result also corroborated with those from previous studies that the WCR midgut had efficient uptake 210-bp long *DvSSJ1*^9^ and 240-bp-long *Snf7*^13^ dsRNAs but did not have efficient uptake 21-bp siRNAs.

**Table 2:** Analysis of WCR mortality fed various lengths of *DvSSJ1* dsRNA.

Treatment description	Mortality (%)	95% Confidence limit for mortality	Fisher's test *P* value for mortality	Mean weight (mg) (95% confidence interval)	Weight range (mg)	*P* value for weight
Bioassay control diet	17.9	6.06–36.9	–	1.28 (0.972–1.58)	0.1–2.5	–
21 bp	31.0	15.3–50.8	0.1988	1.52 (1.19–1.85)	0.2–3.0	0.8561
40 bp	25.9	11.1–46.3	0.3457	0.890 (0.561–1.22)	0.1–2.6	0.0448*
60 bp	50.0	31.3–68.7	0.0101*	0.927 (0.547–1.31)	0.3–2.0	0.0778
80 bp	82.8	64.2–94.2	< 0.0001*	0.300 (-0.358–0.958)	0.1–0.5	0.0044*
100 bp	96.6	82.2–99.9	< 0.0001*	0.100	NA	–
150 bp	96.6	82.2–99.9	< 0.0001*	0.500	NA	–
210 bp	93.1	77.2–99.2	< 0.0001*	0.250 ± 0.212	0.1–0.4	–

A series of seven different lengths of *DvSSJ1* dsRNA was incorporated into a standard artificial diet and WCR mortality was recorded as described in Method.

*A statistically significant difference (*P* value < 0.05) was observed.

#### dsRNA specificity test

To assess the relationship between dsRNA fragment specificity and insecticidal activity, a 21 bp *DvSSJ1* siRNA (TACCGAACCGGATATCAAGGC) was selected based on previous dsRNA feedings^9^ and was flanked by the GFP nucleotide sequence to ensure dsRNA uptake in the WCR gut^9,12^. The purpose was to better understand what level of complementarity was required to elicit biological activity in WCR. Increasing single nucleotide polymorphisms (SNPs) were introduced in various locations within the *DvSSJ1* 21-mer (Supplementary Fig. 9B) and fed to WCR over 14 days to assess biological activity. Acceptable WCR control mortality for these bioassays was considered to be less than or equal to 30%. For the 210 bp GFP sequence 34.5% mortality was observed (Table 3) however, it was not statistically different from the RNase free water control (*P* = 0.2299) and was significantly different when compared with the 21 bp *DvSSJ1* embedded in the GFP nucleotide sequence (*P* = 0.0361). An additional bioassay was conducted to further establish whether the GFP sequence had an effect on WCR mortality or weight, and no activity was observed in this second study (*P* = 1.000 and 0.6546 for mortality and weight, respectively, Supplementary Table 2).

**Table 3:** Analysis of WCR mortality for dsRNA specificity data.

Treatment description	Mortality (%)	95% Confidence limit	FDR adjusted *P* value versus 21 bp *DvSSJ1* in GFP	Fisher's test *P* value versus GFP control
RNAse free H2O	17.2	5.85–35.8	0.0006*	0.2299
GFP control (210 bp GFP)	34.5	17.9–54.3	0.0361*	–
*DvSSJ1* 21 bp embedded within 210 bp GFP	68.0	46.5–85.1	–	0.0281*
*DvSSJ1* 1 bp point mutation (loc 1)	55.2	35.7–73.6	0.4073	0.1864
*DvSSJ1* 2 bp point mutations (locs 1, 3)	20.0	7.71–38.6	0.0008*	0.2516
*DvSSJ1* 3 bp point mutations (locs 1, 3, 5)	23.3	9.93–42.3	0.0018*	0.3985
*DvSSJ1* 4 bp point mutations (locs 1, 3, 5, 7)	17.2	5.85–35.8	0.0006*	0.2299
*DvSSJ1* 5 bp point mutations (locs 1, 3, 5, 7, 9)	17.9	6.06–36.9	0.0006*	0.2299
*DvSSJ1* 1 bp point mutation (loc 11)	16.7	5.64–34.7	0.0006*	0.1432
*DvSSJ1* 210 bp dsRNA	92.9	76.5–99.1	0.0378*	< 0.0001*

Increasing single nucleotide polymorphisms (SNPs) were introduced in various locations (loc) within the *DvSSJ1* 21-mer and fed to WCR over 14 days to assess WCR mortality.

*A statistically significant difference (*P* value < 0.05) was observed.

The 21-bp *DvSSJ1* fragment embedded in the GFP sequence demonstrated the activity to WCR with significant differences from the GFP control (*P* = 0.0361) and the RNase free water control (*P* = 0.0006). Introducing 1 SNP at the first of the 21 base pairs (location 1 or loc 1) did not significantly reduce the biological activity as compared with the exact 21 bp *DvSSJ1* fragment (*P* = 0.4073). Introducing a second SNP at location 3, however, ablated the activity of the *DvSSJ1* fragment (*P* = 0.0008) when compared to the original 21-mer embedded in GFP nucleotide sequence. Similar observations were noted with additional SNPs (3–5) accumulated in the native 21 bp *DvSSJ1* fragment (Table 3). Introducing a single SNP at location 11 also eliminated bioactivity of the dsRNA fragment (*P* = 0.0006). Similar observations were noted for the weight endpoint (Table 4). Therefore, not only does the number of SNPs affect the activity of dsRNA actives, but the relative location of those SNPs is also an important factor in determining activity. The number of SNPs in various locations of the 21nt *DvSSJ1* siRNA were designed in this study based on a preliminary experiment (Supplementary Fig. 8). These results confirm that the siRNA seed region (bases 2–8) and splice site (bases 10–11) are the least tolerant of mismatches because of their active role in gene silencing^18^.

**Table 4:** Summary analysis of WCR weight results for dsRNA specificity data.

Treatment description	Mean weight (mg) (95% confidence interval)	Range (mg)	Adjusted *P* value versus 21 bp *DvSSJ1* in GFP	*P* value versus GFP control
RNAse free H2O	1.93 (1.61–2.26)	0.4–3.5	< 0.0001*	0.9374
GFP control (210 bp GFP)	1.95 (1.59–2.31)	0.2–3.3	< 0.0001*	–
*DvSSJ1* 21 bp embedded within 210 bp GFP	0.375 (-0.183–0.933)	0.1–1.1	–	< 0.0001*
*DvSSJ1* 1 bp point mutation (loc 1)	1.03 (0.593–1.47)	0.3–2.3	0.2387	0.0016*
*DvSSJ1* 2 bp point mutations (locs 1, 3)	1.23 (0.903–1.55)	0.1–2.3	0.0445*	0.0034*
*DvSSJ1* 3 bp point mutations (locs 1, 3, 5)	1.85 (1.52–2.18)	0.3–3.2	< 0.0001*	0.6728
*DvSSJ1* 4 bp point mutations (locs 1, 3, 5, 7)	1.62 (1.29–1.94)	0.2–3.0	0.0012*	0.1727
*DvSSJ1* 5 bp point mutations (locs 1, 3, 5, 7, 9)	2.00 (1.67–2.33)	0.4–2.8	< 0.0001*	0.8486
*DvSSJ1* 1 bp point mutation (loc 11)	1.37 (1.05–1.68)	0.3–3.0	0.0130*	0.0173*
*DvSSJ1* 210 bp dsRNA	1.40 ± 0.707	0.9–1.9	–	–

Increasing single nucleotide polymorphisms (SNPs) were introduced in various locations (loc) within the *DvSSJ1* 21-mer and fed to WCR over 14 days to assess WCR weight.

*A statistically significant difference (*P* value < 0.05) was observed.

### Long dsRNA is the insecticidal active form of RNA molecules *in planta*

**Figure 5:**
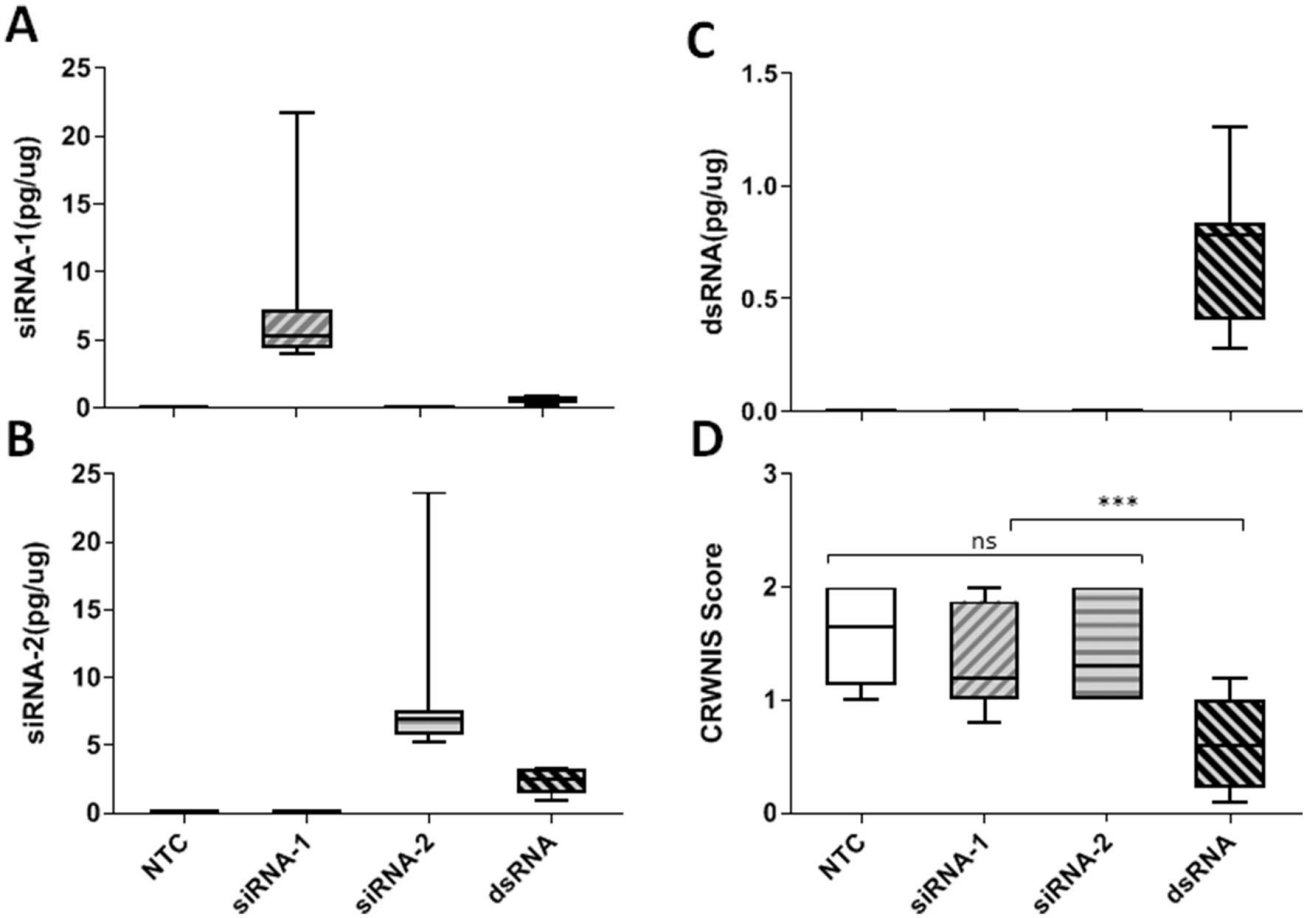
Characterizations of transgenic plants expressing *DvSSJ1* siRNAs and dsRNA. T0 single copy plants were grown in the greenhouse and root tissues were collected for siRNA-1 (**A**), siRNA-2 (**B**) and 210-bp dsRNA (**C**) quantification as described in Method; (**D**) The *DvSSJ1* transgenic lines and non-transgenic control (NTC) were selected for T0 greenhouse assay. Eight plants per *DvSSJ1* dsRNA line and NTC plants were assayed for WCR feeding damage. The CRW nodal injury score (mean ± SD) of dsRNA line was significantly different (*P* value < 0.0004–0.004) between the NTC and siRNA transgenic lines. No significant difference between two small RNA constructs and non-transgenic control (NTC).

To characterize which forms of *DvSSJ1* RNA are the active molecule for mortality of WCR larvae, we designed and generated transgenic plants expressing only 21 bp siRNA of *DvSSJ1* compared with plants expressing both long dsRNA and dicer processed siRNAs^9,12^. Two small RNA sequences were selected based on the results from small RNA profiling of WCR-fed 210-bp *DvSSJ1* dsRNA^9^. *DvSSJ1* 21nt siRNAs were incorporated into the maize miR396h backbone^19,20^ as artificial miRNA (amiRNA) constructs^21^. A construct expressing the 210-bp *DvSSJ1* cassette with the same promoter (UBI) and terminator (PINII) was included for comparison: this cassette produces both long dsRNAs and siRNAs in transgenic tissues. Eight to twelve T0 transgenic lines expressing 21-bp *DvSSJ1* siRNA-1, -2 or dsRNA were selected for greenhouse assay and infested with 1,000 WCR eggs at the V6 (six-leaf) stage. Total RNA was extracted from root tissues for Northern blot and QuantiGene analysis to determine RNA expression in T0 transgenic plants. Both *DvSSJ1* long dsRNA transcripts and dsRNA derived small RNAs (21 to 24-nt RNAs) were analyzed on Northern blots (Fig. 4). QuantiGene Singleplex^22^ siRNA probes (Supplementary Fig. 11) were designed by eBioscience (Thermo Fisher Scientific, Inc.). The probes were validated for sensitivity and specificity for mature siRNA-1 and siRNA-2 in plant samples (Supplementary Table 5). Expression analyses showed that the amiRNA constructs produced 3.6-fold (siRNA-1; 7.45 pg ug^−1^ total RNA) or 12.2-fold (siRNA-2; 8.38 pg ug^−1^ total RNA) higher expression compared to the same siRNAs from the 210 bp dsRNA construct (Fig. 5A, B). *DvSSJ1* long dsRNA transcripts was only detected in transgenic plants expressing the 210-bp *DvSSJ1* cassette (Fig. 5C).

Plants were scored for WCR feeding damage^23^ three weeks after infestation. The average nodal injury score for the transgenic 210-bp *DvSSJ1* events was less than 0.625, which was a significant reduction from the corresponding score of 1.6 for the non-transgenic control (NTC, *P* < 0.0004) and plants expressing only 21-bp small RNAs (*P* < 0.002 and < 0.004, siRNA1 and siRNA 2 respectively; Fig. 5D and Supplementary Table 4). There were no statistically significant differences between the two small RNA constructs and non-transgenic control (NTC). The results presented here suggest that full-length *DvSSJ1* dsRNA can provide WCR protection in transgenic plants. In contrast the transgenic maize plants expressing specific *DvSSJ1* siRNAs do not provide root protection from WCR feeding damage. These results are consistent with both diet-based studies that long dsRNAs are more effective than siRNAs, and previous studies that demonstrate siRNAs were not taken up by WCR midgut^9,13^. These results strongly support long dsRNAs *in planta* are the functional RNA form for control of WCR.

## Conclusions

Plant-delivered long dsRNA is the functional RNA molecule for the protection of transgenic maize from WCR feeding damage. A single nucleotide mutation of 21-bp siRNA can have a significant impact or abolish diet activities against WCR. The molecular target of 210-bp *DvSSJ1* dsRNA is arthropod-specific, has not been described in vertebrates, and is uniquely specific to the *Diabrotica* genus in the Chrysomelidae family of Coleoptera.

## Method and materials

### Plant expression vectors

The transgenic expression cassette (Fig. 1A) is expressed as a transcript that contains two inverted RNA fragments of the smooth septate junction protein 1 (*DvSSJ1*) gene from *Diabrotica virgifera* (WCR)^8^ separated by an intron connector sequence derived from the intron 1 region of the *Zea mays* alcohol dehydrogenase (*zm-Adh1*) gene^24^ to form a hairpin double-stranded RNA. The transcription product of this cassette is intended to knockdown the expression of the smooth septate junction in the mid-gut of the WCR via RNA interference^9^. Expression of the *DvSSJ1* fragments is controlled by a third copy of the *ubi*ZM1 promoter, the 5′ UTR, and intron^25^, in conjunction with the terminator region from the *Zea mays* W64 line 27-kDa gamma-zein (Z27G) gene^26,27^. Two additional terminators are present to prevent transcriptional interference^15^, which includes the terminator region from the *Arabidopsis thaliana* ubiquitin 14 (*UBQ*14*)* gene^28^ and the terminator region from the *Zea mays In2-1* gene^29^. The BSV promoter^30,31^ was also used to express the same *DvSSJ1* cassette. Single copy T0 transformants with a WCR damage score of < 1 were selected^8^ for further characterization at the T1 generation.

### Molecular characterization of *DvSSJ1* transgenic plants

#### Northern analyses and sequencing full-length transcript

Total RNA was isolated using TRIzol Reagent (Invitrogen). Following extraction, the total RNA was visualized on an agarose gel to determine the quality and was quantified on a NanoDrop spectrophotometer (Thermo Fisher Scientific). The mRNA was isolated from total RNA using a FastTrack MAG kit (Invitrogen) and quantified by an Agilent 2,100 bioanalyzer (Agilent Technologies). *DvSSJ1* antisense riboprobe was in vitro transcribed from the *DvSSJ1* PCR product (210 bp) and was labeled with digoxigenin-labeled nucleotides (DIG-11-UTP) according to the procedures provided in the DIG RNA Labeling Kit (Roche). The detailed mRNA northern method was described in the Supplementary Method. Additional northern analyses were conducted with total RNA as described before^8^. One microgram of total RNA was used to generate a cDNA library to sequence the *DvSSJ1* transcript in transgenic plants. Two independent laboratories carried out 3′ and 5′ RACE for sequencing the full-length *DvSSJ1* transcript using a 5′/3′ RACE kit (Roche) and RACE conditions provided in Supplementary Method.

### In situ hybridization (ISH)

For ISH analyses, target probes, preamplifier, amplifier, and label probe (Supplementary Table 4) were designed by Advanced Cell Diagnostics (Hayward, CA) and samples were processed as previously described^32^. Root tissues were fixed in 10% neutral buffered formalin (4% formaldehyde) for 48 to 72 h and processed for paraffin embedding. Sections (4 μm) were processed for RNA in situ hybridization with the BaseScope Detection Kit (RNAscope Red Assay) according to the manufacturer’s standard protocol (Advanced Cell Diagnostics, Hayward, CA). Images were acquired using a Leica Aperio CS2 digital scanner and captured at 40× magnification with a resolution of 0.25 µm pixel^−1^.

### siRNA profile analysis

For small RNA sequencing, 1 μg of RNA was used to generate small RNA libraries using the small RNA-Seq kit (Illumina)^9^. The total clean reads of each sample were normalized into reads per million (RPM) for the expression of *DvSSJ1* siRNAs to determine differentially expressed sRNAs. Trimmed reads 18 to 41 nt in length were aligned to the 210-bp *DvSSJ1* sequence using Bowtie 2^33^. Only perfectly matched reads were included for analysis of sequence length distribution (Supplementary Figure 5). The 18- and 41-nt *DvSSJ1* reads were visualized using Integrative Genomics Viewer software 2.8 (Broad Institute, Cambridge, MA, USA) (https://software.broadinstitute.org/software/igv/). The maize miR168 was determined by searching the miRBase (https://www.mirbase.org/, release 21) and used as a control for quantification and northern analyses of siRNAs (Supplementary Table 4).

### *DvSSJ1* homologous sequence comparison

The sources of *DvSSJ1* 210 bp homologous sequences were identified and selected from published data^8,16^ and listed in Supplementary Table 1. The transcriptomes of six additional insects were assembled as previously described^32^. Briefly, cDNAs prepared from larva were sequenced by Illumina paired-end and 454 Titanium sequencing technologies. De novo transcriptome assemblies were performed using Trinity method^34^. The AlignX tool of Vector NTi 10.3 (Invitrogen) was used to create alignments between the *DvSSJ1* 210-bp sequence and each homologous sequence. Each alignment was inspected to determine the percent identity over the length of the alignment. The longest contiguous match between the *DvSSJ1* fragment and each of the homologous sequences from the various insects was determined by visual inspection. All the data were summarized in Table 1, Supplementary Table 1, and Supplementary Fig. 7.

### Double-stranded RNA production by in vitro transcription

To produce *DvSSJ1* dsRNA, gene-specific primers (Supplementary Table 3) containing T7 RNA polymerase sites (5´d[TAATACGACTCACTATAGGG]3´) at the 5′ end of each primer were used to amplify PCR product which served as the template for dsRNA synthesis by in vitro transcription.

Fragment length samples were treated with RNase A/T1 to remove single-strand T7 sequence from both ends (Supplementary Fig. 9and 10). All dsRNA/RNA samples for bioassays were purified by RNA spin column (Invitrogen), and then quantified by nanodrop 8,000 (Thermo Fisher Scientific).

### WCR bioassay methods

Bioassays were conducted to determine the response of WCR to dsRNA exposure via oral ingestion. WCR eggs were obtained from an internal colony (Johnston, IA) and incubated in an environmental chamber until the eggs hatched. Neonates were used in each bioassay within 24 h of hatching. All bioassays were conducted in 24-well Falcon culture plates. Stonefly Heliothis Diet (SHD, Ward’s Science, Rochester NY) comprised the base dry diet for WCR. A proprietary mixture of additional ingredients was added to SHD to stimulate feeding and/or allow for the biological maturation of WCR. In internal testing, this diet typically generates less than 30% mortality in the negative control, similar to other WCR laboratory diets^17^. On Day 0 of each respective bioassay, the dry diet was mixed with RNase free water (negative control) or a liquid test substance of interest to achieve the desired concentration. Approximately 300 µl (~ 300 mg) of freshly prepared diet was dispensed into wells of the bioassay plates. One WCR neonate was placed in each well. Each plate was sealed with heat-sealing film, and two small holes were poked over each well to allow for ventilation. Bioassays were conducted in an environmental chamber set at 21 °C, 65% relative humidity, and continuous dark for a total of 14 days. Every 3 to 4 days, new bioassay plates were prepared with fresh diet as described for Day 0, and living WCR were transferred to the new plates and missing or dead organisms were recorded. After 14 days, each bioassay was complete, final mortality was assessed, and surviving organisms were individually weighed. Organisms recorded as missing from a well or lost in the transfer, or wells containing more than one organism, were excluded from statistical analysis.

### Artificial miRNA expression vectors and transformation

Two 21-mer’s, siRNA-1 and -2 of *DvSSJ1* were selected based on previous dsRNA-feeding data, which showed higher accumulation in WCR midgut^9^, and were designed for expressing 21-bp siRNAs according to the rules for artificial microRNA design^20,35^. The 21-bp siRNAs were incorporated into the zma-miR396h backbone^19,20^ to compare with the dsRNA 210-bp fragment of the *DvSSJ1* gene^8^. The silencing cassettes (Fig. 4) consisted of the maize ubiquitin promoter, maize ubiquitin intron 1^36^, siRNA unit or two 210 bp stretches of *DvSSJ1* and an intervening truncated maize ADH intron1, designed to support assembly into a dsRNA hairpin, and the PIN II terminator^37^. The *DvSSJ1* constructs were transformed via *Agrobacterium tumefaciens* into a commercial maize elite-inbred line, PHR03^38^. T0 maize transformants were screened by qPCR analyses^39^ and single-copy T-DNA integration events were used for further characterization. In the T0 greenhouse assay, 10–12 independent events per each construct (siRNA), eight *DvSSJ1* 210-bp dsRNA events (T0 plants) and non-transformed PHR03 control (NTC) were infested with 1,000 WCR eggs at the V6 (six-leaf) stage and evaluated for WCR damage^23^ and to support the expression of RNA’s analyses^8^.

For expression analyses, total RNA was extracted using the mirVana miRNA Isolation kit (Life Technologies, Carlsbad, CA) from T0 transgenic maize plants at the leaf stage V6-V7. Ten µg of total RNA was fractionated on a 1.5% denaturing formaldehyde gel for long dsRNA northern analysis. For siRNA northern blot analysis, 20 µg of total RNA was fractionated on a 15% Criterion TBE-Urea Gel (Bio-Rad, Hercules, CA). Hybridization and washing conditions were as described previously^8^.

### Quantigene analyses for siRNA and dsRNA quantification in plants

Quantification of dsRNA transcripts was conducted as previously reported^32^ and siRNAs were detected by a luminometer (Promega Glomax) using a modified method^40^. Target specific probes (Supplementary Table 3) were designed to quantify *DvSSJ1* siRNAs using a QuantiGene miRNA 2.0 assay (Supplementary Fig. 11). RNA oligo duplexes of siRNA-1 and -2 were used to generate a standard curve for the quantification and ZM-miR168 oligo duplex was included as an internal control to normalize sample variations. Aliquots of each plant RNA sample were diluted to 6.25 ng μl^−1^ using TE buffer. Each reaction comprised 125 ng of total RNA in a total volume of 20 μl. Standard curves comprised duplexed RNA oligo’s diluted with 10 ng μl^−1^ carrier yeast RNA. Standard curves covered six-points of each targeted duplex ranging from 10 pg – 0.0032 pg, at fivefold dilutions per point. Samples, controls, and curves were all run in triplicate as technical repeats. Standard curves were employed to calculate pg of siRNA per reaction which was further extrapolated to pg of siRNA per μg of total RNA. Hybridization, signal amplification, and data acquisition were carried out in a standard 96-well plate following the procedure described in Supplementary Method.

### Statistical analysis

The Statistical analysis for this paper was generated using SAS software, Version 9.4. Copyright [2019] SAS Institute Inc. SAS and all other SAS Institute Inc. product or service names are registered trademarks or trademarks of SAS Institute Inc., Cary, NC, USA.

### WCR diet bioassays

For each treatment mortality was estimated with exact (Clopper-Pearson) 95% confidence intervals. Fisher’s exact test (SAS PROC MULTTEST) was then used to determine if the mortality rate of each treatment was greater than the mortality associated with the bioassay control.

For weight, SAS PROC GLIMMIX was used to conduct a linear model analysis to generate estimated means, 95% confidence intervals and the statistical comparisons between each treatment and the bioassay control. Error was initially assumed both independent and identically distributed and later confirmed by visual inspection of the residuals from the fitted model.

*dsRNA Fragment Length data:* Treatments with two or fewer surviving insects were not included in the analysis. For both mortality and weight, statistical significance was established if the *P* value was < 0.05. No multiplicity adjustment was used because the risk of falsely declaring that a treatment does not significantly differ from the control was considered of greatest concern.

*dsRNA Specificity data:* For comparison against the 21 bp RNA embedded within the 210 bp GFP, a multiple comparison adjustment was conducted using the false discovery rate (FDR) method^41,42^. In this instance falsely declaring a treatment differed from the 21 bp RNA embedded within the 210 GFP was considered of greatest concern, thus the FDR adjustment was appropriate. For comparisons against the 210 bp GFP control no FDR adjustment was employed as falsely declaring no significant difference was considered of greatest concern thus the FDR adjustment was not used. For weight, multiple comparison adjustments were conducted as noted above for the mortality endpoint except using Dunnett's method^40^ instead of FDR.

### Plant bioassays

Mean values and standard deviations were calculated by linear regression using Minitab 15 statistical software. The difference in WCR nodal injury score was tested by a one-way analysis of variance taking the phenotype and the presence or absence of the transgene as the sources of variation. Tukey’s family error rate was chosen for one-way multiple comparisons with a *P* value level of significance equal to 0.05.

## Supplementary information

Supplementary Figures and Tables
