# Author Correction: Characterization of *DvSSJ1* transcripts targeting the smooth septate junction (SSJ) of western corn rootworm (*Diabrotica virgifera virgifera*)

**DOI:** 10.1038/s41598-020-75942-5

**Published:** 2020-11-04

**Authors:** Xu Hu, Chad J. Boeckman, Bin Cong, Joe P. Steimel, Nina M. Richtman, Kristine Sturtz, Yiwei Wang, Carl A. Walker, Jiaming Yin, Anita Unger, Caitlin Farris, Albert L. Lu

**Affiliations:** Corteva Agriscience, 7300 NW 62nd Ave, Johnston, IA 50131 USA

Correction to: *Scientific Reports* https://doi.org/10.1038/s41598-020-68014-1, published online 07 July 2020

The original version of this Article contained a typographical error in the spelling of the author Carl A. Walker, which was incorrectly given as Carl L. Walker. As a result, the Author Contribution statement contained an error where the initials of Carl A. Walker were incorrectly given as C.L.W.. This has now been corrected in the PDF and HTML versions of the Article, as well as the accompanying Supplementary Information file.

Additionally, this Article contained an error in the Method and materials section under the subheading ‘Plant bioassays’.

“The difference in leaf number was tested by a one-way analysis of variance taking the phenotype and the presence or absence of the transgene as the sources of variation.”

now reads:

“The difference in WCR nodal injury score was tested by a one-way analysis of variance taking the phenotype and the presence or absence of the transgene as the sources of variation.”

This error has been corrected in the PDF and HTML versions of the Article.

