# Supplementary material for: Characterization of DvSSJ1 transcripts targeting the smooth septate junction (SSJ) of western corn rootworm (Diabrotica virgifera virgifera)
Source: Sci Rep. 2020 Jul 7;10:11139. doi: 10.1038/s41598-020-68014-1 (PMC7341793; doi:10.1038/s41598-020-68014-1)
Supplement: Supplementary file 1 — Supplementary Figures and Tables [file 41598_2020_68014_MOESM1_ESM.pdf]

Characterization of *DvSSJ1* Transcripts Targeting the Smooth Septate Junction (SSJ) of Western Corn Rootworm (*Diabrotica virgifera virgifera*)

Xu Hu, Chad J. Boeckman, Bin Cong, Joe P. Steimel, Nina M. Richan, Kristine Sturtz, Yiwei Wang, Carl A. Walker, Jiaming Yin, Anita Unger, Caitlin Farris, and Albert L. Lu

Corteva Agriscience, Johnston, IA, USA.

*Corresponding Authors: Xu Hu, Corteva Agriscience, 7300 NW 62nd Ave., Johnston, IA 50131, USA

Telephone: 1-(515)-535-2105

E-mail:xu.hu@corteva.com

Supplementary Figure and Table

A

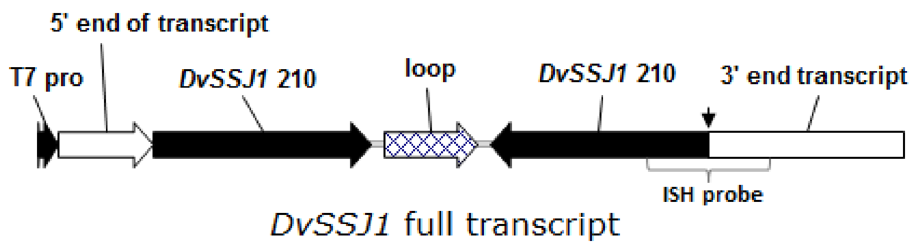

B

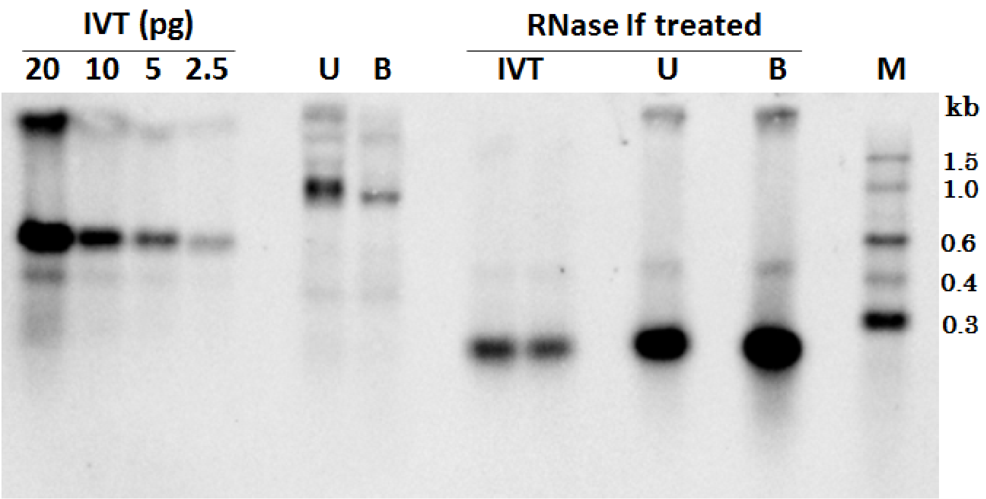

Supplementary Fig 1. Sequence analysis of UBI::*DvSSJ1* full transcript expressed in transgenic plants

(A). Diagram of UBI::*DvSSJ1* full transcript. T7 promoter was added to generate DNA template for *in vitro* transcription of *DvSSJ1* full transcript, which was used as a Quantigene standard for quantification of *DvSSJ1* dsRNA in transgenic plants. The junction region between, *DvSSJ1* and the 3' end sequence was used as a probe to visualize *DvSSJ1* transcript *in planta* by *in situ* hybridization (ISH). (B). Northern analysis of UBI::*DvSSJ1* full transcript expressed in transgenic plants. Total RNA samples from plants (20 ug per well) under the control of UBI (U) or BSV (B) promoters and dsRNA *DvSSJ1* with loop region made by IVT (50 and 25 pg; 590 nt with loop) were treated with or without RNase I_f (NEB) resulting in the 210 bp fragment. All samples were run on a 2.5% agarose gel containing 6% formaldehyde in 1x MOPS.

A

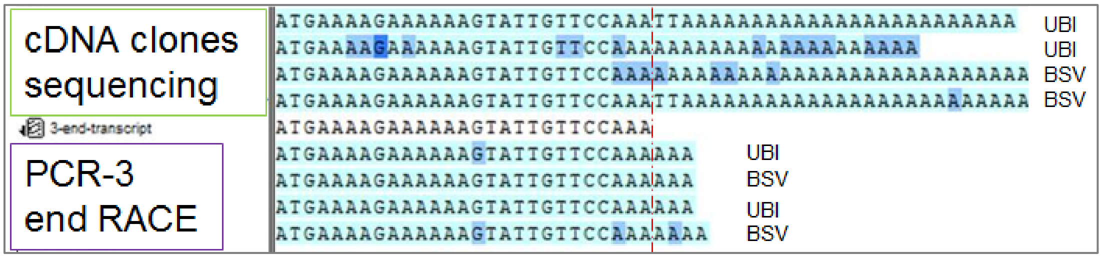

B

```
5'-end:    1   tccccaacctcgtgttgttcggagcgnnnnnnnnnnnnaccagatctcccccaaatccac 60
               ||||||||||||||||||||||||||            ||||||||||||||||||||||
S94464.1: 902  tccccaacctcgtgttgttcggagcgcacacacacacaaccagatctcccccaaatccac 961

5'-end:    61  ccgtcggcacctccgctcaaggt 84
               ||||||||||||||||||||||||
S94464.1: 962  ccgtcggcacctccgctcaaggt 985
```

C

```
3'-end:    21  ccggcggtgtcccccactgaagaaactatgtgctgtagtatagccgctggctagctagct 80
               ||||||||||||||||||||||||||||||||||||||||||||||||||||||||||||
X53514.1: 2362 ccggcggtgtcccccactgaagaaactatgtgctgtagtatagccgctggctagctagct 2421

3'-end:    81  agttgagtcatttagcggcgatgattgagtaataatgtgtcacgcatcaccatgcatggg 140
               ||||||||||||||||||||||||||||||||||||||||||||||||||    ||||||
X53514.1: 2422 agttgagtcatttagcggcgatgattgagtaataatgtgtcacgcatcac----catggg 2477

3'-end:    141 tggcagtctcagtgtgagcaatgacctgaatgaacaattgaaatgaaaagaaaaaagtat 200
               ||||||| ||||||||||||||||||||||||||||||||||||||||||||||||||||
X53514.1: 2478 tggcagtgtcagtgtgagcaatgacctgaatgaacaattgaaatgaaaagaaaaaagtat 2537

3'-end:    201 tgttccaaa 209
               |||||||||
X53514.1: 2538 tgttccaaa 2546
```

Supplementary Fig 2. Sequences of both *DvSSJ1* cDNA clones or PCR RACE products

(A). Sequence analysis of 3' end of *DvSSJ1* transcripts under the control of UBI or BSV promoters were carried out by both cDNA library construction or PCR-based RACE methods. Sequencing of both cDNA library clones and 3' end RACE demonstrated the same 3' ending for both UBI or BSV constructs; *DvSSJ1* transcription ends 166 bp downstream of GZ-W64A terminator; Two constructs showed the same ending location (red vertical line) followed by poly-A tail; (B) 5' end of sequence alignments for *DvSSJ1* transcript sequences, which are partially matched to 5UTR of maize polyubiquitin (S94464.1) and, (C) 3' end of *DvSSJ1* transcript aligned to terminator of maize zein Zc2 gene (X53514.1) as part of the construct elements. The AlignX tool of Vector NTi 10.3 (Invitrogen) was used to create alignments or sequence comparison.

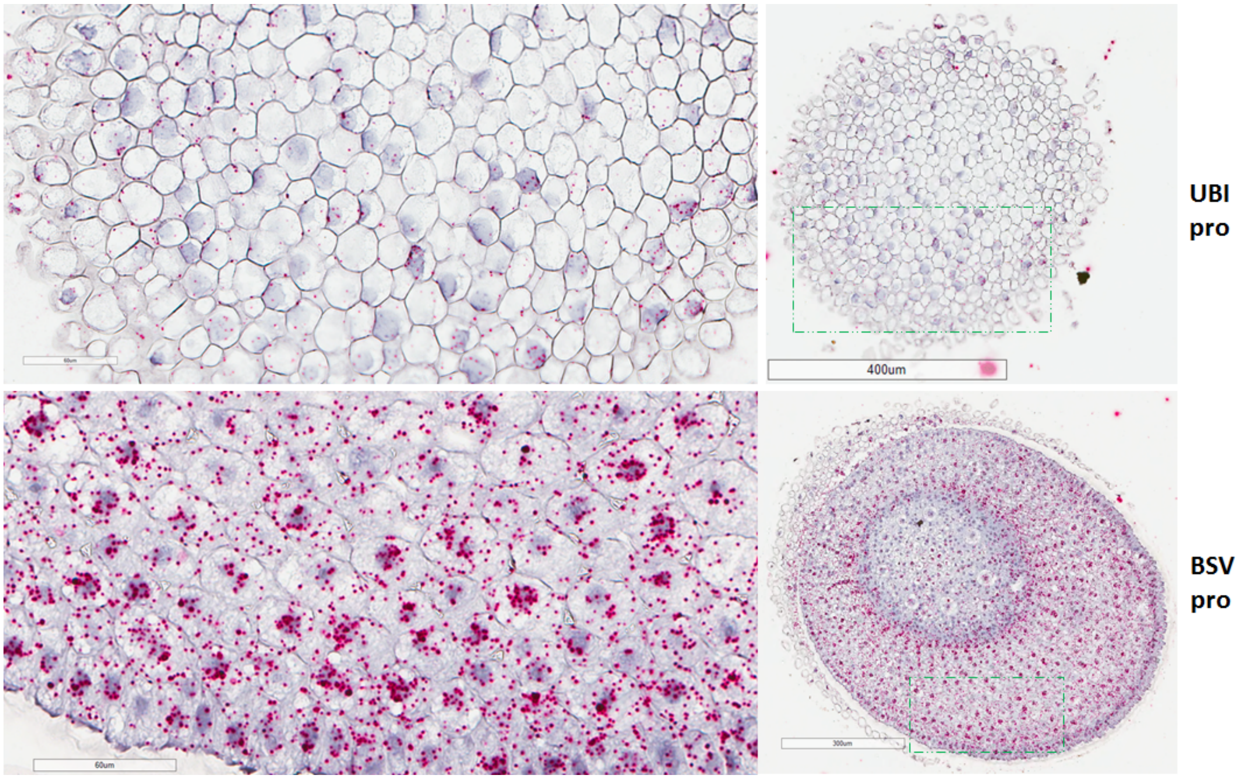

Supplementary Fig 3. Cross-sections of transgenic root tips expressing *DvSSJ1*

Transgenic root samples were collected at V6 stage in the greenhouse and hybridized with *DvSSJ1probe Dvv-ssj1*-P (supplmentary table 4). A single red dot represents one *DvSSJ1* transcript in the root cell. Scale bar in right panel=400 um; 300um; green boxes were enlarged and shown in left panel (Scale bar=60 um).

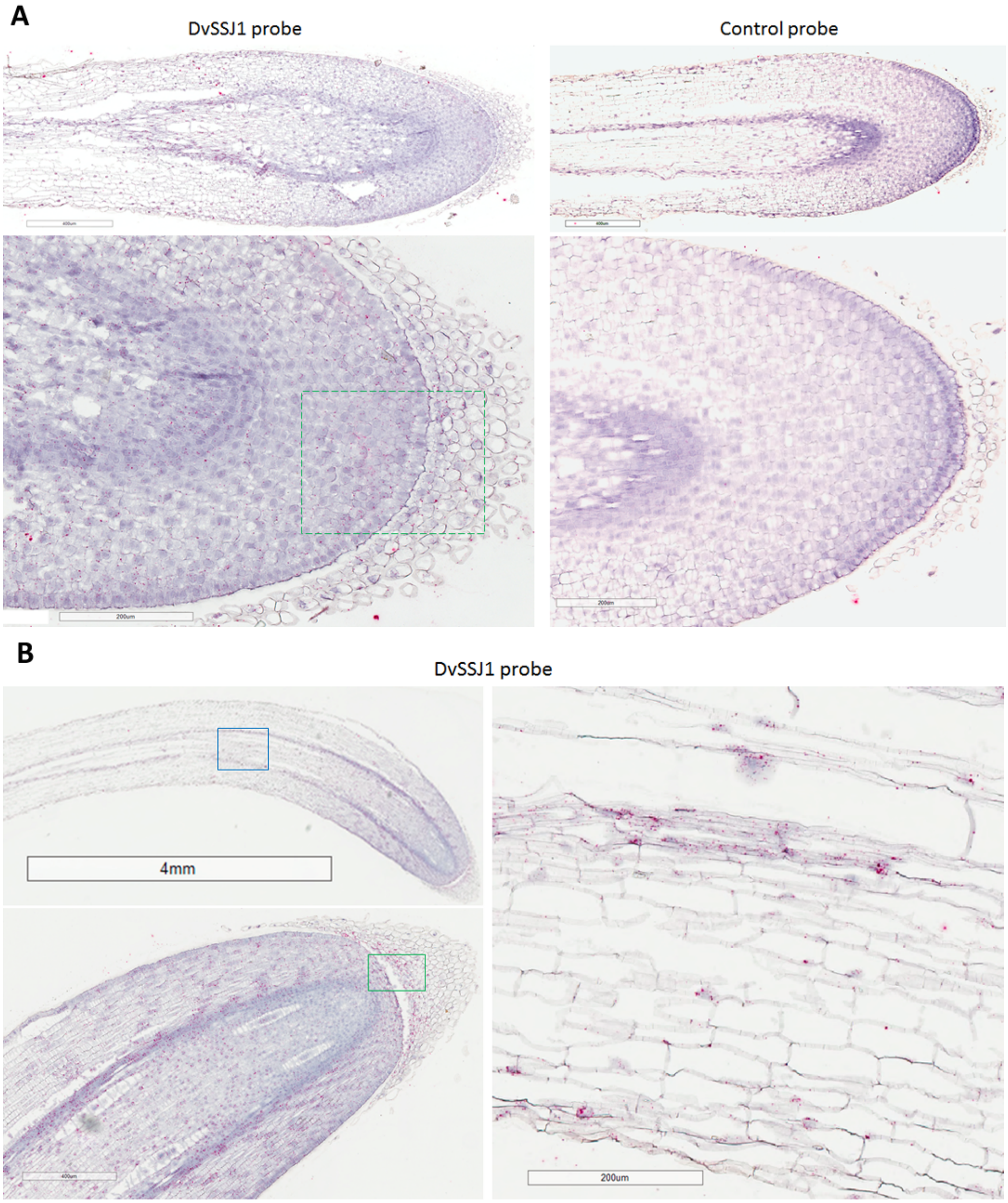

Supplementary Fig 4. Longitudinal sessions of root tips expressing *DvSSJ1*

Transgenic root samples of UBI (A) and BSV (B) promoter-driven *DvSSJ1* plants were hybridized with the *DvSSJ1* probe *Dvv-ssj1-P*, as well as negative control probe (dapB), as described in Methods and Supplmentary table 4. Green boxes in root tips were presented in the main text (Fig 2) and the blue box, in the mature root, is shown in the right panel (Scale bar=200 um).

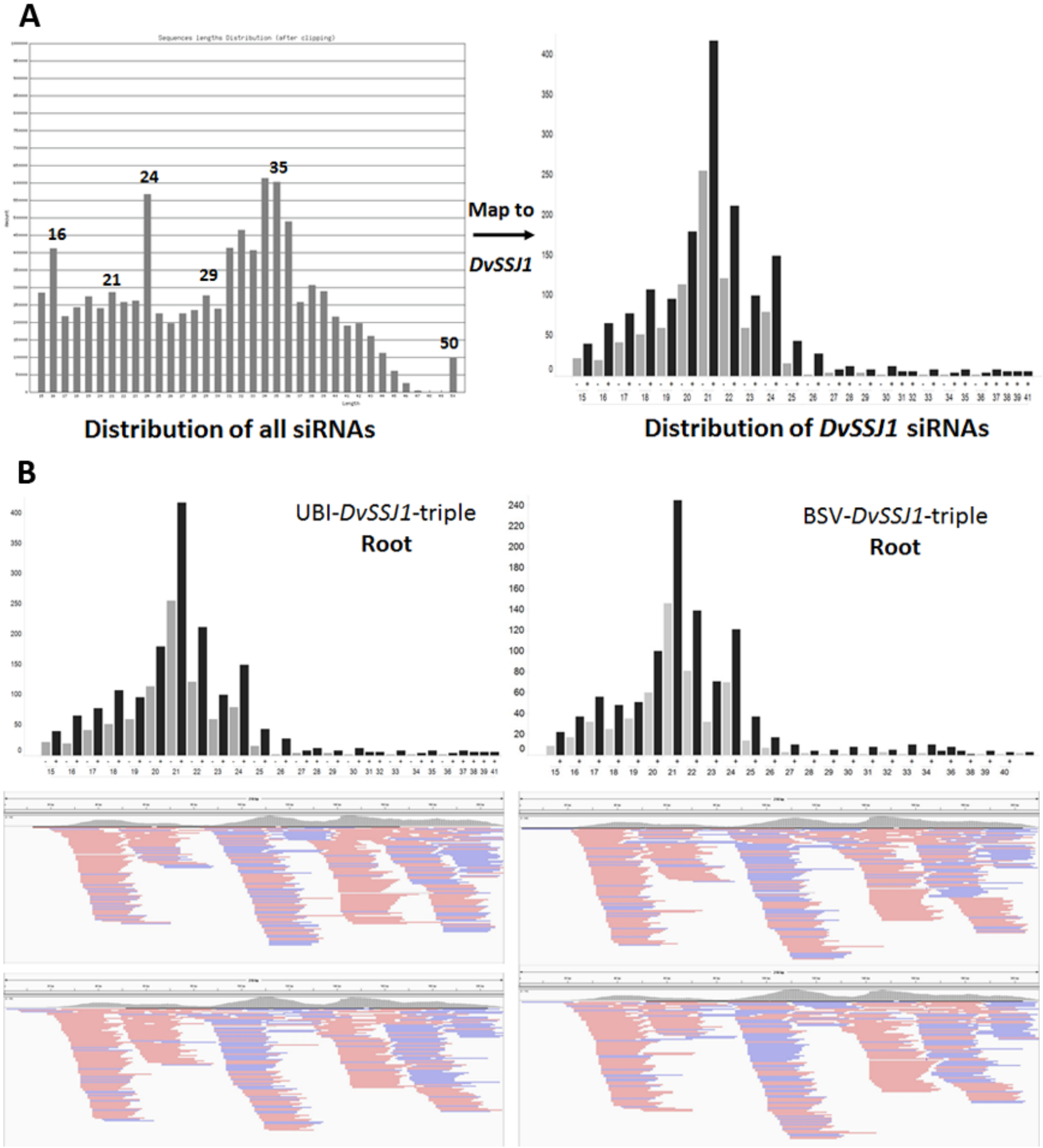

Supplementary Fig 5. **Analyses of *DvSSJ1* siRNA expression in transgenic plants**

(A).siRNA sequencing revealed the sequence length, position, strand, abundance, and abundance_percentage information for *DvSSJ1* specific reads. The abundance percentage is a simple statistic calculated as = (read abundance * 100 / total abundance) for reads that mapped in that respective sample; the mapping percentages to *DvSSJ1* 210-bp were quite low (~ 0.1-0.5% of total siRNAs) for selected samples. (B). siRNA analyses of root samples (two replicates) from UBI and BSV promoters. *DvSSJ1* siRNA reads were visualized using Integrative Genomics Viewer software 2.8 (Broad Institute, Cambridge, MA, USA) (http://software.broadinstitute.org/software/igv/).

A

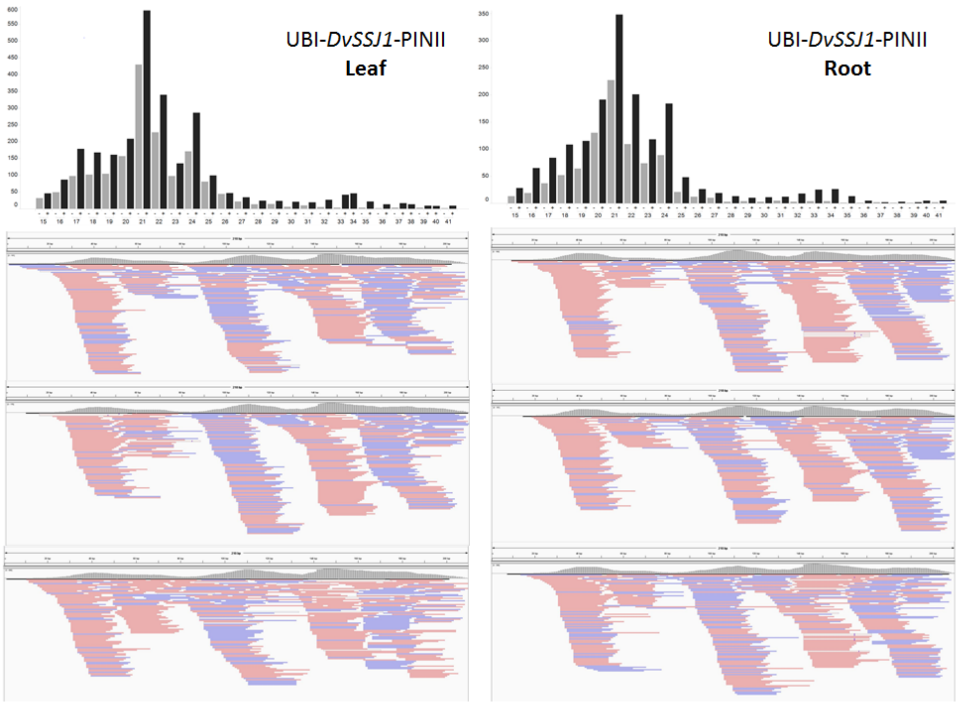

B

Average percentage of top *DvSSJ1* siRNAs in transgenic plants				
**Constructs**	**A**	**B**	**C**	
**Promoter**	**UBI Pro**	**BSV Pro**	**UBI Pro**	**UBI Pro**
**Terminator**	**Triple**	**Triple**	**PIN II**	**PIN II**
**Tissue**	**Root**	**Root**	**Leaf**	**Root**
**replicates**	**2**	**2**	**3**	**3**
AGGAACCTGGAATCTAAACGA	25.33	23.70	27.38	18.33
ACCTGGAATCTAAACGAAGAA	7.22	4.43	7.70	5.85
AGGAACCTGGAATCTAAACGAA	9.21	9.37	6.81	5.32
AGGAACCTGGAATCTAAACGAAGA	4.06	5.37	6.47	6.24
GAGGAACCTGGAATCTAAACGA	2.84	2.86	2.66	2.53
TCTGCATCGGGATTTTTTTCT	0.99	1.63	2.67	0.90

Supplementary Fig 6. Comparison of siRNA analyses between root and leaf tissues

Samples were collected from plants with the construct configuerations UBI::*DvSSJ1*:: PIN II terminator and BSV::*DvSSJ1*:: PIN II terminator (A). Three replicates per tissue type were included in this study. *DvSSJ1* siRNA reads were visualized using Integrative Genomics Viewer software 2.8 (Broad Institute, Cambridge, MA, USA) (http://software.broadinstitute.org/software/igv/). (B) Summary of the average percentage of top *DvSSJ1* siRNA's from different constructs and tissue types. Percent of individual siRNA was calculated by specific siRNA count dividing by total *DvSSJ1* siRNA counts, then times 100.

```
                 1                                                                   70
SSJ1       (1)  --MTSIETVGTIVLKLLKLVINLICLILYRTGYQGYFLGVGGTWNLNEEKNPDAEIVASGVFVGFMIYTF
 CDM       (1)  --MVSVQTIATITVKVFKIVLNIIILVLYRTGYNGEFLGVGGTWNLNEEKNPDAEIVASGVIVGYLIYTL
 MBD       (1)  --MTSVETIGAIAIRIVKLVINFIIIMYRTGNHGGFLGVGGTWNLNEVKSADVEIVASGVFVGYFIFTA
 MWM       (1)  --MTSIETVGALILKILKLVLNLIILILYRTGFSGGFLGVGGTWNLNEEKNPDAEIVASGIFVGYFIYTS
 PLD       (1)  --MVSVQTIATIVVKVFKIVLNIVILVLYRTGYNGEFLGVGGTWNLNEEKNPDAEIVASGVIVGYLIYTL
 RVB       (1)  MAASSIMTVGSIFLKIFKLVLNLIILVLYRVGFHGGFLGVGGTWNLNEEKNPDAEIVASGVFVGYFIYTT
 SWM       (1)  --MTSIETVGALILKILKLVLNLIILILYRTGYGGGFLGVGGTWNLNEEKNPDAEIVASGVFVGYFIYTC
                71                                                                  140
SSJ1      (69)  VSLISLCFASGDHKTTFTDILMNIVGIFMWIAVGATALHYWLGYLSEYKYTTIDSERQVGLALGAMCIIN
 CDM      (69)  VQCITFLFGTTEHKRALSEIVMNFVGVFLWIAVGAVALHYWGGYQGEHQYQFVFAEKQVGLAVGALCVIQ
 MBD      (69)  VSLMSLLFGTRDNKVYFTDLIMNIIGVLMWLIVGGIAVHYWNGYIDEQKYQEVSSERTVGLIMGALCIIT
 MWM      (69)  VSIISLCFASGDHKNTFTDILMNIIGVFLWVAVGATALHYWSGYLLEHKYQTTASEREVGLAMGALCILS
 PLD      (69)  VQIVTFLFGTTEHKRALSEIVMNFIGVFMWIAVGAVALHYWGGYQGEHQFQFVFAEKQVGLAVGALCVIQ
 RVB      (71)  VSLISYCFSTNDHKHSFTDILMNIIGTMMWLATGATALHYWTGYLSEYKFTATASERQVGLALGSLCVIS
 SWM      (69)  VSIISLCFATADHKNTFTDILMNIIGVFLWVAVGATALHYWSGYLVEHKYLTSASEREVGLAMGSLCILS
                141                        167
SSJ1     (139)  GAVYLVDGVLSAIFILKAKMQ------
 CDM     (139)  GAVYLLDTALSVIHFTKEM--------
 MBD     (139)  AALYLIDSVVSVLFVVREKLNGQFA--
 MWM     (139)  GAAYLVDTVLSVVFVIKAKL-------
 PLD     (139)  GAVYLLDTALSVIHFTKEM--------
 RVB     (141)  GATYLLDSVLSVIHVIRSHQAKEEDDE
 SWM     (139)  GAAYLVDTVLAIIFVIKAKM-------
```

Supplementary Fig 7. Protein alignment of *DVSSJ1* and its homologs from six new insects

Western corn rootworm *DvSSJ1* gene (KU562965) was used for tblastx search transcriptome assembly of seven additional insect species and their respective SSJ1 protein sequence. *DvSSJ1* (SSJ1) was used to perform alignments with the six additional target proteins. *Zophobas morio* Super worm (SWM); *Tenebrio molitor* Mealworm (MWM); *Dalotia coriaria* Rove beetle (RVB); *Cryptolaemus montrouzieri* Mealy bug destroyer (MBD); *Vanessa cardui* Painted Lady (PLD); *Cydia pomonella* Codling Moth (CDM). Nucleotide sequences of six insects were used for sequence analyses (Table 1 and Supplementary Table 1). The AlignX tool of Vector NTi 10.3 (Invitrogen) was used to create alignments.

A.

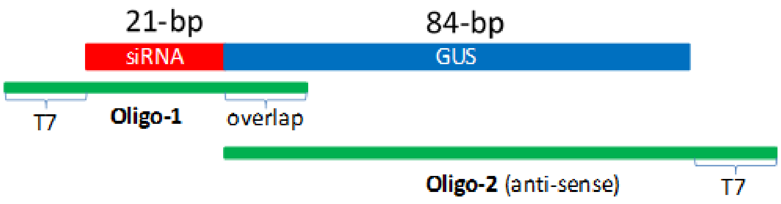

B.

Name	siRNA mutation changes	# of SNP	Ave Score	1	2	3	4	5	6	7	8
siRNA	**TACCGAACCGGATATCAAGGC**	0	2.5	3	3	2	2	3	3	2	2
mut-1	TACCGAACCGGATATCAAGGA	1	2	2	2	2	2	2	2	2	2
mut-2	aACCGAACCGGATATCAAGGA	2	1.875	2	2	1	2	2	2	2	2
mut-3	atCCGAACCGGATATCAAGGA	3	1.875	2	2	2	2	1	2	2	2
mut-4	atgCGAACCGGATATCAAGGA	4	1.125	1	1	1	2	1	0	2	1
mut-5	atggGAACCGGATATCAAGGA	5	0.875	1	2	1	0	0	2	0	1
mut-6	atggcAACCGGATATCAAGGA	6	0.375	1	0	0	0	0	1	0	1
mut-7	atggctACCGGATATCAAGGA	7	0	0	0	0	0	0	0	0	0
mut-8	atggcttCCGGATATCAAGGA	8	0	0	0	0	0	0	0	0	0
mut-9	atggcttgCGGATATCAAGGA	9	0	0	0	0	0	0	0	0	0
mut-10	TtCCGAACCGGATATCAAGGA	2	1.375	1	1	2	2	2	1	1	1
mut-11	TAgCGAACCGGATATCAAGGA	2	1.25	1	2	1	1	1	2	1	1
mut-12	TACCGtACCGGATATCAAGGA	2	1.625	2	1	1	2	2	1	2	2
mut-13	TACCGAACgGGATATCAAGGA	2	1.25	1	2	2	0	1	1	1	2
mut-14	TACCGAACCcGtTATCAAGGA	3	0	0	0	0	0	0	0	0	0
mut-15	TACCGAACCcGtTtTCAAGGA	4	0	0	0	0	0	0	0	0	0
mut-16	TACCGAACCGGtTATCAAGGA	2	1.125	2	1	1	1	0	1	1	2
mut-17	TACCGAACCGGATAaCtAccA	5	0	0	0	0	0	0	0	0	0
mut-18	TACCGAACCGGATATCtAccA	4	1.571		2	1	2	1	2	1	2
mut-19	TACCGAACCGGATATCAAccA	3	1		0	2	0	1	1	1	2
mut-20	TACCGAACCGGATATCAAGcA	2	2.125	2	3	2	2	2	2	2	2
GUS	TCTTTATACCGAAAGGTTGGG	13	0.125	1	0	0	0	0	0	0	0
Water		n/a	0	0	0	0	0	0	0	0	0
Water		n/a	0		0	0	0	0	0	0	0

Supplementary Fig 8. The preliminary experiment of siRNA mutation and dsRNA for diet assay against WCR. All dsRNA samples were produced by IVT method using PCR-amplified DNA templates. (**A**). The β-Glucuronidase (GUS) sequence was used as a carrier (84-bp) or control (21-bp). Single nucleotide polymorphisms (SNPs, red color) were introduced in various locations within the *DvSSJ1* siRNA. Double-stranded RNA was incorporated into WCR diet at a final concentration of 100 ng μl^{-1} in a 96 well plate format. Insects were scored for mortality and stunting (**B**) after 7 days and an average score was assigned based on 8 observations (replicates) for each dsRNA target as described before[1]. Seed region is in green color.

A. Information for *DvSSJ1* fragment length-activity study

Treatment number	Treatment description	Length (bp)	Pictorial representation	IVT Template
1	Negative control (H_2O)	n/a		
2	GFP	210		PCR
3	Positive control (*DvSSJ1* dsRNA)	210		PCR
4	test	150		PCR
5	test	100		PCR
6	test	80		PCR
7	test	60		Oligo synthesis
8	test	40		Oligo synthesis
9	test	21		Oligo synthesis

B. *DvSSJ1* 21-bp sequence specificity study

Treatment number	Treatment description	% mismatch[a]	Length (bp)	Pictorial representation	21 bp sequence (mutation in red)[b]
1	Negative control (H_2O)	--			
2	GFP	--	210		n/a
3	Positive control (*DvSSJ1* dsRNA)	--	210		TACCGAACCGGATATCAAGGC
4	100% match	0	210		TACCGAACCGGATATCAAGGC
5	1bp mutation	4.8	210		AACCGAACCGGATATCAAGGC
6	2bp mutation	9.5	210		AAGCGAACCGGATATCAAGGC
7	3bp mutation	14.3	210		AAGCCAACCGGATATCAAGGC
8	4bp mutation	19.1	210		AAGCCATCCGGATATCAAGGC
9	5bp mutation	23.8	210		AAGCCATCGGGATATCAAGGC
10	1bp mutation	4.8	210		TACCGAACCGCATATCAAGGC

a: % mismatch to *DvSSJ1* 21 bp insert; b: mutated nucleotide (s) shows in the red and yellow box indicates the region of mutation (s) in 21-bp siRNA; underline indicates siRNA seed region

Supplementary Fig 9. Information for *DvSSJ1* fragment length (top) and 21-bp siRNA specificity (bottom) studies. All dsRNA samples were produced by IVT method using PCR-amplified template or synthesized DNA oligo's as templates (A); 210-bp fragment (B; 21-bp *DvSSJ1* siRNA flanked by GFP: Supplementary Table 3) were cloned into *E. coli* vector (pUC57) and DNA templates were made by PCR with same primer pair. Fragment length samples were treated with RNase A/T1 to remove single-strand T7 sequence from both ends. siRNA seed region is underlined. *DvSSJ1* siRNA (TACCGAACCGGATATCAAGGC) was selected based on previous dsRNA feeding [2].

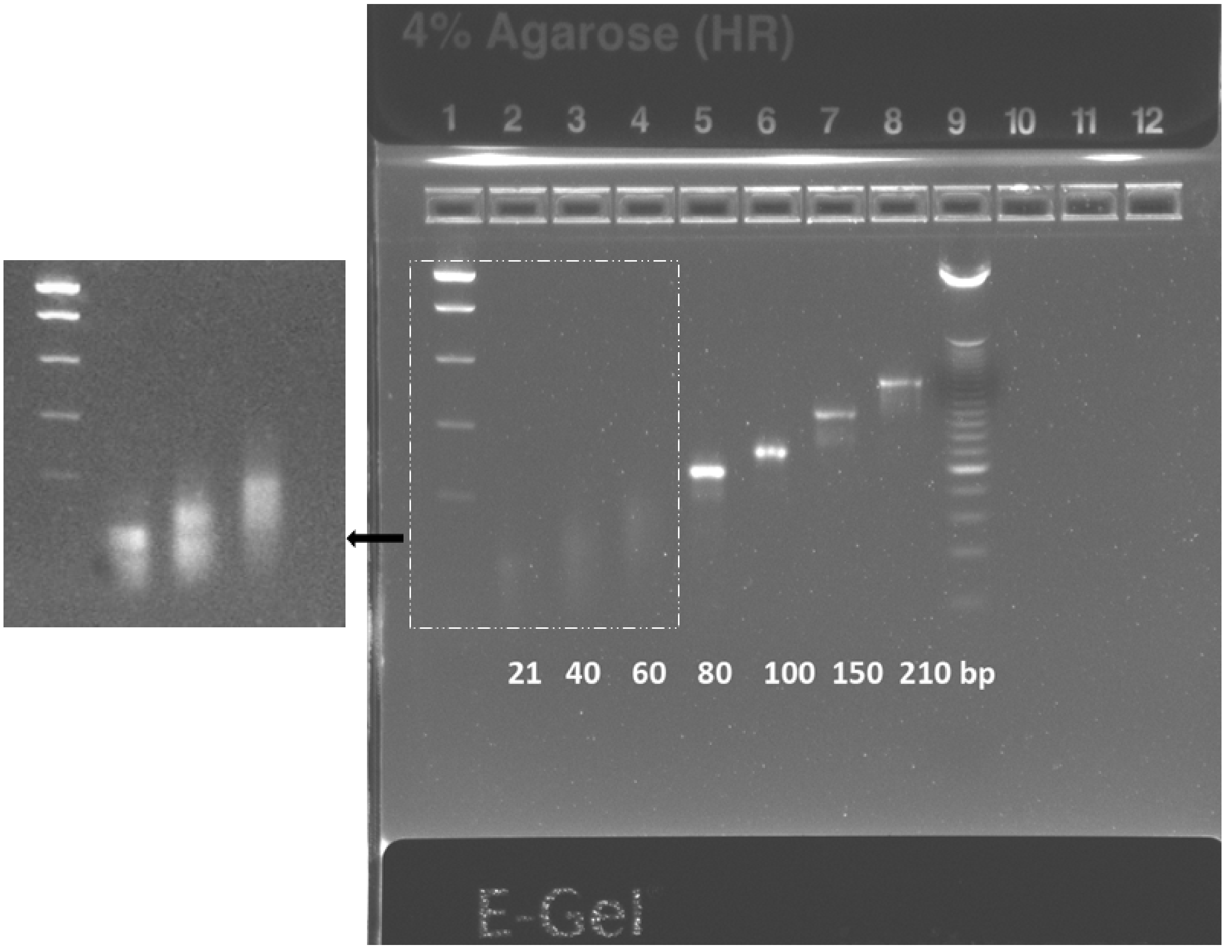

Supplementary Fig 10. DsRNA of *DvSSJ1* different sizes on 4% agarose gel

Samples were prepared via IVT and treated with RNase A/T1 to remove single-strand T7 sequence from both ends. Spin-column purified dsRNA were run on 4% E-gel (Invitrogen) and then quantified by nanodrop 8000 for WCR bioassay. Lane 9 is 25 bp size marker.

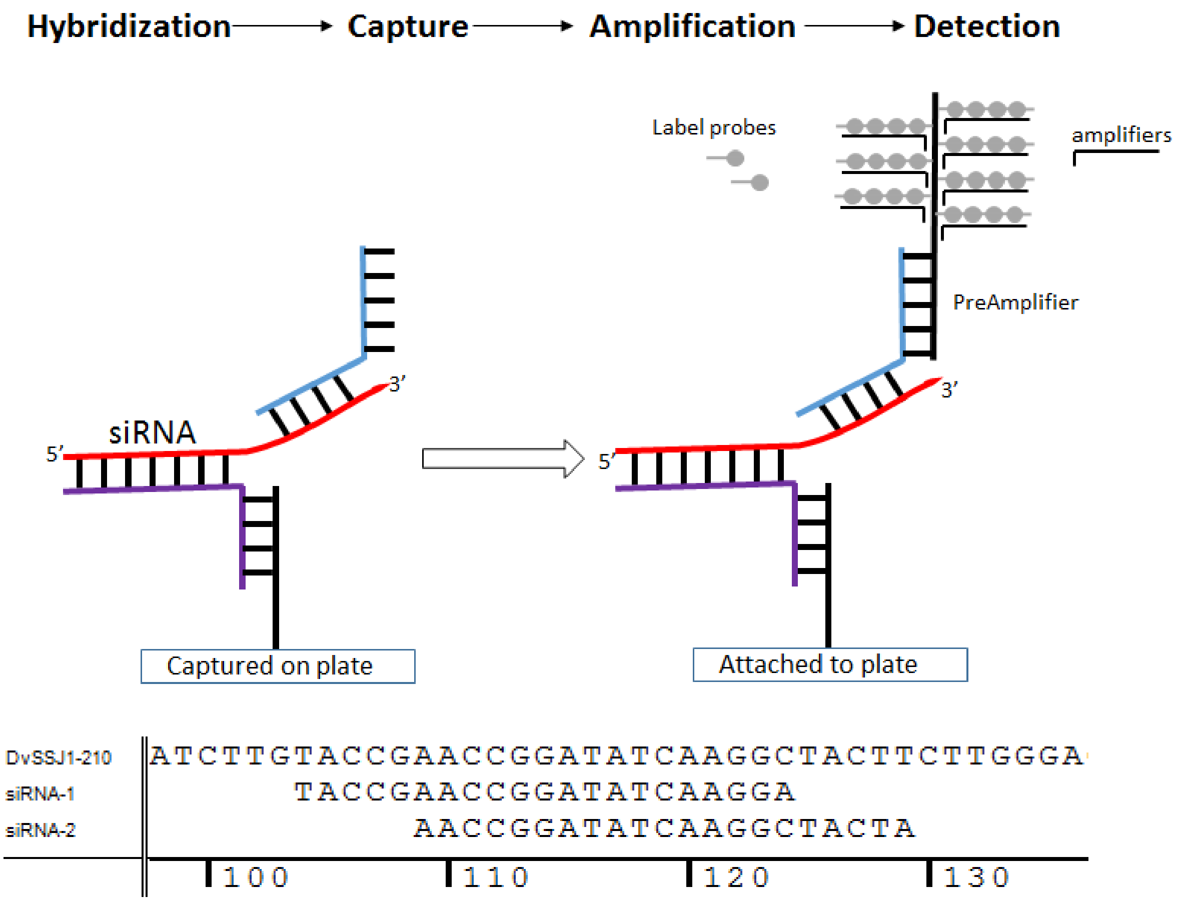

Supplementary Fig 11. Detection of *DvSSJ1* siRNAs using QuantiGene miRNA assay

A diagram illustrates QuantiGene Singleplex Assay workflow (Thermo Fisher Scientific). QuantiGene Singleplex Probe Sets (siRNA-1, -2 and zm-miRNA168) was used for quantitation of target-specific RNAs in transgenic tissues (pg siRNA per ug total RNA). Plant extracts were directly processed and quantified according to the QuantiGene 2.0 miRNA (Singleplex) Assay User Manual. RNA sense-antisense oligo duplexes of siRNA-1 and -2 were used to generate a standard curve for quantification and ZM-miR168 oligo duplex was included as an internal control to normalize sample variations. The detailed procedure is described in the Supplementary Method.

Supplementary Table 1: List of sequence sources of *DvSSJ1* homologous corresponding to 210-bp fragment

Order	Common Name	**Scentific name**	Sequence Sources	Distance[a]
Coleoptera	Western corn rootworm	*Diabrotica virgifera*	Hu et al [1]	0
	Northern corn rootworm	*Diabrotica barberi*	Hu et al [1]	0.027
	Southern corn rootworm	*Diabrotica undecimpunctata*	Hu et al [1]	0.068
	Crucifer flea beetle	*Phyllotreta cruciferae*	WO2016043960	0.233
	Striped flea beetle	*Phyllotreta striolata*	WO2016043960	0.255
	Colorado potato beetle	*Leptinotarsa decemlineata*	Hu et al [1]	0.282
	Red flour beetle	*Tribolium castaneum*	Hu et al [1]	0.358
	Super worm	*Zophobas morio*	this study[c]	0.369
	Mexican bean beetle	*Epilachna varivestis*	Hu et al [1]	0.475
	Mealworm	*Tenebrio molitor*	this study[c]	0.420
	Rove beetle	*Dalotia coriaria*	this study[c]	0.437
	Mealy bug destroyer	*Cryptolaemus montrouzieri*	this study[c]	0.496
	Pink spotted lady beetle	*Coleomegilla maculata*	WO2016043960	0.517
Lepidoptera	Painted Lady	*Vanessa cardui*	this study[c]	0.489
	European corn borer	*Ostrinia nubilalis*	WO2016043960	0.520
	Fall armyworm	*Spodoptera frugiperda*	WO2016043960	0.504
	Codling Moth	*Cydia pomonella*	this study[c]	0.551
	Corn earworm	*Helicoverpa zea*	WO2016043960	0.531
Hymenoptera	Honey bee	*Apis mellifera*	Hu et al [1]	0.416
Hemiptera	Insidious flower bug	*Orius insidiosus*	Hu et al [1]	0.510

a: Estimates of Evolutionary Divergence between Sequences of *DvSSJ1* 210 bp fragment and the corresponding homolog from different insect species; The number of base substitutions per site between sequences of *DvSSJ1* 210 bp fragment and the corresponding homolog are shown. Analyses were conducted using the Maximum Composite Likelihood model [3]. The analysis involved 20 nucleotide sequences. Codon positions included were 1st+2nd+3rd+Noncoding. All positions containing gaps and missing data were eliminated. There were a total of 185 positions in the final dataset. Evolutionary analyses were conducted in MEGA7 [4].

Supplementary Table 2. Summary Analysis of Mortality Results for GFP follow-up data

Treatment Description	Mortality (%)	95% Confidence Limit	Fisher's Test P-Value for mortality	Mean weight (mg) (95% Confidence Interval)	Weight Range (mg)	P-Value for weight
Bioassay Control - RNAse free H2O	6.67	0.818 - 22.1	1.0000	1.88 (1.57 - 2.18)	0.3 - 3.2	0.6546
Green Fluorescent Protein Control (GFP)	3.33	0.0844 - 17.2	--	1.78 (1.48 - 2.08)	0.5 - 3.9	--
DvSSJ1 210bp dsRNA	96.7	82.8 - 99.9	<0.0001*	0.500	NA	--

*A statistically significant difference (P-Value < 0.05) was observed

For the follow-up experiment with GFP, a similar analysis was conducted as for the siRNA specificity data without the multiplicity adjustment. Mortality was estimated for each treatment with exact (Clopper-Pearson) 95% confidence intervals. Fisher's exact test (SAS PROC MULTTEST) was used to compare the mortality rate of each treatment to the mortality rate observed with GFP control.

For weight, SAS PROC GLIMMIX was again used to conduct a linear model analysis to generate estimated means, 95% confidence intervals and the statistical comparisons between each treatment to the GFP control. An error was initially assumed both independent and identically distributed and later confirmed by visual inspection of the residuals from the fitted model. A significant difference was identified if the P-value was < 0.05.

Supplementary Table 3. PCR primers to generate DNA template for IVT

**PCR primers to amplify DNA template from 210-bp DvSSJ1 fragment**		nt or bp	
ssj-210-F	TAATACGACTCACTATAGGGATAATAAGTTCGATTTTTTACGAAAATG	48	forward primer with T7
ssj-210-R	TAATACGACTCACTATAGGGTACGAATACGCCGGAAGC	38	reverse primer with T7
ssj-150-F	TAATACGACTCACTATAGGGAGACTGTGGGGACCATTGTCCTG	43	forward primer with T7
ssj-150-R	TAATACGACTCACTATAGGGCAATTTCTGCATCGGGAT	38	reverse primer with T7
ssj-100-F	TAATACGACTCACTATAGGGATTGCTGAAGTTGGTGATCA	40	forward primer with T7
ssj-100-R	TAATACGACTCACTATAGGGTTCGTTTAGATTCCAGGT	38	reverse primer with T7
ssj-80-F	TAATACGACTCACTATAGGGTTGGTGATCAATTTGATATG	40	forward primer with T7
ssj-80-R	TAATACGACTCACTATAGGGTTCCAGGTTCCTCCTACT	38	reverse primer with T7
ssj-60-F	TAATACGACTCACTATAGGGATTTGATATGTCTCATCTTGTACC	44	forward primer with T7
ssj-60-R	TAATACGACTCACTATAGGGCTCCTACTCCCAAGAAG	37	reverse primer with T7
**DNA oligos for double-strand DNA template**			
ssj-60-t7	TAATACGACTCACTATAGGGATTTGATATGTCTCATCTTGTACCGAACCGG ATATCAAGGCTACTTCTTGGGAGTAGGAG	80	sense strand
ssj-60as-t7	TAATACGACTCACTATAGGGCTCCTACTCCCAAGAAGTAGCCTTGATATCC GGTTCGGTACAAGATGAGACATATCAAAT	80	antisense strand
ssj-40-t7	TAATACGACTCACTATAGGGTCTCATCTTGTACCGAACCGGATATCAAGG CTACTTCTTG	60	sense strand
ssj-40as-t7	TAATACGACTCACTATAGGGCAAGAAGTAGCCTTGATATCCGGTTCGGTA CAAGATGAGA	60	antisense strand
ssj-21-t7	TAATACGACTCACTATAGGGTACCGAACCGGATATCAAGGC	41	sense strand
ssj-21as-t7	TAATACGACTCACTATAGGGGCCTTGATATCCGGTTCGGTA	41	antisense strand
**PCR primer to amplify DNA template from *DvSSJ1*-GFP in pUC57 plasmid**			
forward	TAATACGACTCACTATAGGGTCATCTGCACCACCGGCAAG	40	siRNA specificity study
reverse	TAATACGACTCACTATAGGGCCTCGAACTTCACCTCGGCG	40	siRNA specificity study
**Tempate DNA for DvSSJ1 mismatch study**			
GFP-210	TCATCTGCACCACCGGCAAGCTGCCTGTGCCCTGGCCCACCCTGGTGAC CACCCTGAGCTACGGCGTGCAGTGCTTCTCACGCTACCCCGATCACATGA AGCA**gcacgacttcttcaagagcgc**CATGCCTGAGGGCTACATCCAGGAGCGCA CCATCTTCTTCGAGGATGACGGCAACTACAAGTCGCGCGCCGAGGTGAA GTTCGAGG	210	210 bp of AcGFP; 21mer in the middle (red) was replaced with dvssj1 siRNA
GUS	GATGCTCCATCACTTCCTGATTATTGACCCACACTTTG CCGTAATGAGTGACCGCATCGAAACGCAGCACGATACG CTGGCCTG	84	84 bp of Escherichia coli beta-D-glucuronidase gene

Supplementary Table 4. Probe information for expression analyses

**Probe for northern analyses**		size (nt or bp)	
DvSSJ1 probe	ATAATAAGTTCGATTTTTACGAAAATGACAAGTATCGAGACTGTGGGGACCATTGTCCTGAAA<br>TTGCTGAAGTTGGTGATCAATTTGATATGTCTCATCTTGTACCGAACCGGATATCAAGGCTACT<br>TCTTGGGAGTAGGAGGAACCTGGAATCTAAACGAAGAAAAAAATCCCGATGCAGAAATTGTG<br>GCTTCCGGCGTATTCGTAGG	210	northern analysis*
***in situ* hybridization probes**			
Dvv-ssj1-P	TTCCTCCTACTCCCAAGAAGTAGCCTTGATATCCGGTTCGGTACAAGATGAGACATATCAAATT<br>GATCACCAACTTCAGCAATTTCAGGACAATGGTCCCCACAGTCTCGATACTTGTCATTTTCGTA<br>AAAAATCGAACTTATTATCCTAGTTAGTTAGGTTGTAGTTAAACAGCCCCCTCCGGCGGTGTCC<br>CCCACTGAAGAAACTATGTGCTGTAGTATAGCCGCTGGCTAGCTAGCTAGTTGAGTCATTTAG<br>CGGCGATGATTGAGTAATAATGTGTCACGCATCACCATGCATGGGTGGCAGTCTCAGTGTGA<br>GCAATGACCTGAATGAACAATTGAAATGAAAAGAAAA	354	targeting 5' end 160 bp dvssj1 hairpin and 194 bp of terminator region
Dvv-R10	CTTTCTGATTTTTGACAGCTTCTATAGAAGTTTATCAAGATGTTGATGCCAAAAAAGAATAGAGT<br>ATGTATTTACGAATACCTCTTCAAAGAGGGAGTCATGGTAGCTAAAAAAGATTACCATGCCCC<br>AAAACACCTCGAACTAGAAACTATCCCTAACCTTCAAGTAATTAAGGCTTTACAATCACTTAAAT<br>CAAAAGGTTACGTAAAGGAACAATTCGCCTGGAGGCATTATTATTGGTATTTGACTAACTCTG<br>GCATCGAATACCTCCGCACATTCTTACACTTACCTGGAGAAATTGTCCCATCTACCTTGAAACG<br>CCCAGCAAGGACAGAAACCACCCGTCCTAGACCAGCTGCTCTCAGATCTGAGACATCTAAAC<br>CTTCAGAAGACCGTGCAGGATACAGAAGGACTCCTGGAGGCCCTGGAGCTGACAAGAAAGC<br>TGATGTTGGTCCAGGAACTGGAGATGTTGAGTTCAGGCAAGGATTCGGACGTGGACGGGCA<br>CCACAATAAATTTATTGATAAGTTAATTTTTATAAATTGATCAGCCAAT	553	ribosomal protein S10 (RPS10) targeting 107-552 region of KU756281.1
Negative control	GTGTTCGTTCTGCCAATTTAACAGCTTCCTGCCCCATTCTTC	42	*Bacillus subtilis* dihydrodipicolinate reductase (dapB) gene targeting 821-862 region
**siRNA northern analysis**			
Exiqon LNA 5' Biotin-labelled DNA probes for target specific siRNA northern			
siRNA-1	TCCTTGATATCCGGTTCGGTA	21	WCR siRNA-1
siRNA-2	TAGTAGCCTTGATATCCGGTT	21	WCR siRNA-2
zma-MIR168A	GTCCCGATCTGCACCAAGCGA	21	internal control
**siRNA QuantiGene analysis**			
Probe design			
siRNA-1	TCCTTGATATCCGGTTCGGTA	21	WCR siRNA-1
siRNA-2	TAGTAGCCTTGATATCCGGTT	21	WCR siRNA-2
zma-MIR168A	UCGCUUGGUGCAGAUCGGGAC	21	internal control
RNA oligo duplexs	Generating standard curve or internal control		
siRNA-1	UCCUUGAUAUCCGGUUCGGUA	21	WCR siRNA-1
siRNA-2	UAGUAGCCUUGAUAUCCGGUU	21	WCR siRNA-2
zma-MIR168A	UCGCUUGGUGCAGAUCGGGAC	21	internal control

DvSSJ1 antisense probe was labeled with DIG as described in Supplementary method and hybridized with mRNAs from T1 plant tissues; T0 dsRNA northern (Fig 4) was conducted as previously described[1].

Supplementary Table 5. Detection and differentiation of siRNA-1, and siRNA-2 in plant root samples* by QuantiGene miRNA Singleplex Assay

Sample ID	Construct ID	Probe	Source	Luminescence
Sample-A	siRNA-1	siRNA-1	Total RNA	4773
Sample-A	siRNA-1	siRNA-1	Total RNA	5224
Sample-A	siRNA-1	siRNA-1	Total RNA	4990
Sample-B	siRNA-1	siRNA-1	Total RNA	4754
Sample-B	siRNA-1	siRNA-1	Total RNA	5043
Sample-B	siRNA-1	siRNA-1	Total RNA	5469
Sample-C	siRNA-2	siRNA-1	Total RNA	-15
Sample-C	siRNA-2	siRNA-1	Total RNA	-13
Sample-C	siRNA-2	siRNA-1	Total RNA	-9
Sample-D	siRNA-2	siRNA-1	Total RNA	-9
Sample-D	siRNA-2	siRNA-1	Total RNA	-3
Sample-D	siRNA-2	siRNA-1	Total RNA	-4
Sample-E	dsRNA	siRNA-1	Total RNA	357
Sample-E	dsRNA	siRNA-1	Total RNA	353
Sample-E	dsRNA	siRNA-1	Total RNA	355
Sample-F	dsRNA	siRNA-1	Total RNA	239
Sample-F	dsRNA	siRNA-1	Total RNA	300
Sample-F	dsRNA	siRNA-1	Total RNA	194
NTC	n/a	siRNA-1	Total RNA	-3
NTC	n/a	siRNA-1	Total RNA	-2
NTC	n/a	siRNA-1	Total RNA	5
Water	n/a	siRNA-1	n/a	13
Water	n/a	siRNA-1	n/a	29
siRNA-1 .002	n/a	siRNA-1	Duplexed Oligo	12176
siRNA-1 .0004	n/a	siRNA-1	Duplexed Oligo	2842
siRNA-1 .00008	n/a	siRNA-1	Duplexed Oligo	506
siRNA-1 .000016	n/a	siRNA-1	Duplexed Oligo	144
siRNA-1 .0000032	n/a	siRNA-1	Duplexed Oligo	23

Sample ID	Construct ID	Probe	Source	Luminescence
Sample-A	siRNA-1	siRNA-2	Total RNA	-50
Sample-A	siRNA-1	siRNA-2	Total RNA	-46
Sample-A	siRNA-1	siRNA-2	Total RNA	-38
Sample-B	siRNA-1	siRNA-2	Total RNA	-42
Sample-B	siRNA-1	siRNA-2	Total RNA	-38
Sample-B	siRNA-1	siRNA-2	Total RNA	-36
Sample-C	siRNA-2	siRNA-2	Total RNA	2694
Sample-C	siRNA-2	siRNA-2	Total RNA	3260
Sample-C	siRNA-2	siRNA-2	Total RNA	3154
Sample-D	siRNA-2	siRNA-2	Total RNA	4855
Sample-D	siRNA-2	siRNA-2	Total RNA	5169
Sample-D	siRNA-2	siRNA-2	Total RNA	4671
Sample-E	dsRNA	siRNA-2	Total RNA	698
Sample-E	dsRNA	siRNA-2	Total RNA	647
Sample-E	dsRNA	siRNA-2	Total RNA	728
Sample-F	dsRNA	siRNA-2	Total RNA	366
Sample-F	dsRNA	siRNA-2	Total RNA	338
Sample-F	dsRNA	siRNA-2	Total RNA	313
NTC	n/a	siRNA-2	Total RNA	-25
NTC	n/a	siRNA-2	Total RNA	32
NTC	n/a	siRNA-2	Total RNA	-8
Water	n/a	siRNA-2	n/a	-35
Water	n/a	siRNA-2	n/a	-42
siRNA-2 .002	n/a	siRNA-2	Duplexed Oligo	9041
siRNA-2 .0004	n/a	siRNA-2	Duplexed Oligo	1961
siRNA-2 .00008	n/a	siRNA-2	Duplexed Oligo	378
siRNA-2 .000016	n/a	siRNA-2	Duplexed Oligo	50
siRNA-2 .0000032	n/a	siRNA-2	Duplexed Oligo	-14

*Two representative samples from each construct expressing *DvSSJ1* siRNA-1, siRNA-2 or dsRNA were used for QuantiGene analysis. Luminescence was recorded by Glomax luminometer (Promega) as described in Supplementary Method. A serial dilution of siRNA duplex oligo (ng per reaction) was used. NTC=Non-transgenic control

Supplementary Method

Confirmation of *DvSSJ1* transcript in T1 transgenic plants

RNA extraction: Leaf and root of vegetative stage 5-9, and kernel (reproductive stage 3-4) tissues were harvested from the *DvSSJ1* transgenic plants under the control of UBI and BSV promoters and the non-transgenic control plants (NTC). The plant samples were ground to a fine powder in liquid nitrogen for RNA isolation. Total RNA was isolated using TRIzol Reagent (Invitrogen).

3' RACE and PCR: One microgram of total RNA extracted from each transgenic and control maize plant was treated with DNase I (Amplification grade, Invitrogen) prior to cDNA synthesis. First-strand cDNA was produced with SuperScript III reverse transcriptase (Invitrogen) with primer 17-O-5963 [GACTCGAGTCGACATCGA (T)18] based on the manufacturer's instructions. The reverse transcribed first-strand cDNA then served as a template for two rounds of PCR amplification (first PCR and nested PCR) using Phusion High-Fidelity DNA polymerase (Thermo Fisher Scientific). Primer sets 17-O-5967 [GGAAGCCACAATTTCTGCAT] and 17-O-5965 [CGAACCGGATATCAAGGCTA] and 17-O-5968 [ACAATGGTCCCCACAGTCTC] and 17-O-5965 were used for the first PCR and the nested PCR, respectively. The nested PCR products were purified with a PCR Cleanup Kit (Qiagen) and the purified PCR products were directly sequenced by Eurofins Genomics (Luxembourg). In addition, one microgram of total RNA isolated from root tissue (UBI promoter) was used for repeating the 3' RACE experiment by using the GeneRacer Kit (Invitrogen). The treated total RNA with DNase I was reverse-transcribed using GeneRacer Oligo dT primer based on the manufacturer's instructions. RT minus reaction was set up containing all ingredients as in the reverse transcription reaction but SuperScript III. First PCR to amplify the 3' cDNA ends was performed using a primer set: 17-O-5983 [AATTGAGAATTCGATATCAG] and GeneRacer 3' primer. Nested PCR was performed to increase the specificity of the PCR product using another primer set: 17-O-5983 and GeneRacer 3' Nested PCR primer. The PCR Products were purified and cloned, and then sequenced using M13 forward and reverse primers.

5' RACE and PCR: The same GeenRacer kit was used to amplify the 5' cDNA ends based on the manual. Two micrograms of total RNA from root tissue (UBI promoter) was dephosphorylated with CIP, and then phenol extracted, and ethanol precipitated. The treated RNA was further de-capped and purified prior to GeneRacer RNA oligo ligated to full-length mRNA. *DvSSJ1* specific primer 17-O-5976 was used for reverse transcription. First PCR was performed using GeneRacer 5' primer and 17-O-5985 [AGGTTCCTCCTACTCCCAAGAAGTA] primer. Nested PCR was performed using GeneRacer 5' nested primer and 17-O-5986 [ACATATCAAATTGATCACCAACTTCAGC]. Both PCRs used Phusion High-Fidelity DNA polymerase. The PCR product was excised from an agarose gel, then purified and cloned for sequencing.

Northern analyses of long dsRNAs from plant samples

Leaf (V5-9), root (V5-V9) and kernel (R3-4) tissues were harvested from the BSV and UBI transgenic plants and the non-transgenic control plants (NTC). The plant samples were ground to a fine powder in liquid nitrogen for RNA isolation. The total RNA was visualized on an agarose gel to determine the quality and was quantified on a NanoDrop spectrophotometer (Thermo Fisher Scientific). mRNA was isolated from total RNA using a FastTrack MAG kit (Invitrogen) and quantified by an Agilent 2100 bioanalyzer (Agilent Technologies).

Denatured mRNA, total RNA, total RNA treated with RNase I_f (New England Biolabs), or *DvSSJ1 in vitro* transcript samples were subjected to standard agarose-formaldehyde gel electrophoresis to separate the transcripts by size (2.5% agarose gel containing 6% formaldehyde in 1x MOPS). Also loaded on the gel were DIG-labeled molecular weight markers (Roche). The separated RNA transcripts on the agarose-formaldehyde gel were transferred to a nylon membrane in 20x SSC buffer using the method as described for the TURBO-BLOTTER™ Rapid Downward Transfer System (Whatman, Inc.). Following the transfer, the RNA was bound to the membrane by UV crosslinking using Stratalinker (Stratagene).

DvSSJ1 antisense riboprobe was *in vitro* transcribed from the *DvSSJ1* PCR product (210bp; Supplementary Table 4) with a T7 promoter sequence included in one of the primers as a template. The probe was labeled with digoxigenin-labeled nucleotides (DIG-11-UTP) into the new transcribed product according to the procedures provided in the DIG RNA Labeling Kit (Roche). Labeled *DvSSJ1* antisense probe was hybridized to the RNA on the nylon membranes for detection of the transcripts using the procedures essentially as described for DIG Easy Hyb solution (Roche). The membrane was prehybridized in DIG Easy Hybridization buffer at 68°C for 6 hours at a slow speed in an orbital incubator. The labeled probe was denatured at 98°C for 10 min, and then added to fresh DIG Easy Hybridization buffer (preheated to 68°C) at 68°C in the orbital incubator for overnight. After hybridization, the membrane was washed twice in 2 x SSC, 0.1% SDS solution at room temperature on an orbital shaker for 10 min each, then followed by washing twice in 0.1 SSC, 0.1% SDS at 68°C for 30 min each in the orbital incubator.

DvSSJ1 antisense probes hybridized to RNA bound to the nylon membrane after stringent washes were visualized using the CDP-Star Chemiluminescent Nucleic Acid Detection System with DIG Wash and Block Buffer Set based on the manufactures' instructions (Roche). Blots were exposed to X-ray film to detect hybridized fragments and to visualize DIG-labeled molecular weight standards. Images were also captured with a Luminescent Image Analyzer LAS-4000 (Fujifilm Medical Systems).

Quantigene analyses for siRNA quantification in plants

Target hybridization

Aliquots of each total RNA sample were diluted to 6.25 ng/µl using TE buffer. Each QuantiGene miRNA assay reaction comprised of 125 ng of total RNA in a total volume of 20 µl. Appropriate negative controls comprised of both non-transgenic maize RNA and homogenization solution (ThermoFisher QG0517) in respective reactions. Standard curves comprised of duplexed RNA oligo's (IDT) diluted with 10 ng μl^{-1} carrier yeast RNA. Standard curves covered six-points of each targeted duplex ranging from 10pg – 0.0032pg per reaction, at 5-fold dilutions per point. Samples, controls and

curves were all run in triplicate as technical reps. Prepared samples, controls and duplexed oligo's were aliquoted into a standard 96 well PCR plate, containing the following master mix of the kit provided and assay-specific components per well: 33.3 µl lysis buffer, 1.0 µl blocking reagent, 0.3 µl capture extender (CE), 0.3ul label extender (LE), and 45.1 µl nuclease-free water. The contents were mixed, spun at 240 X g for 20 sec, denatured for 3 min at 95°C and cooled to 46°C. The entire contents were transferred to a room temperature capture plate. The sealed capture plate containing the combined total volume of 100ul/well was spun at 240 X g for 20 sec. and placed in a 46°C stationary incubator for 16-20 Hrs.

Signal amplification and data acquisition

200 µl of the kit provided 1X wash buffer was added to the capture plate(s) and the contents forcibly expelled. Each plate was inverted and tapped onto a clean paper towel. This process was repeated two additional times with 300 µl of 1X wash buffer. After the last wash, inverted plates were spun onto a paper towel at 240 X g for 1 min. The 2.0 PreAmp was diluted with the amplifier/label probe diluent per kit instructions and 100ul added per well and the plate(s) resealed and incubated at 46°C for 1 hour. The above process was repeated for the sequential hybridization of the 2.0 amplifier and the alkaline phosphatase label probe. After the last spin dry, 100ul of room temperature 2.0 chemiluminescent substrate was added per well, the plate(s) resealed and incubated at RT for 5 minutes. Unsealed plates were read in a Glomax luminometer (Promega) set at a 0.2 second integration time.

1 Hu, X. *et al.* Discovery of midgut genes for the RNA interference control of corn rootworm. *Scientific reports* **6**, 30542, doi:10.1038/srep30542 (2016).

2 Hu, X. *et al.* Molecular characterization of the insecticidal activity of double-stranded RNA targeting the smooth septate junction of western corn rootworm (Diabrotica virgifera virgifera). *PLOS ONE* **14**, e0210491, doi:10.1371/journal.pone.0210491 (2019).

3 Tamura, K., Nei, M. & Kumar, S. Prospects for inferring very large phylogenies by using the neighbor-joining method. *Proceedings of the National Academy of Sciences of the United States of America* **101**, 11030-11035, doi:10.1073/pnas.0404206101 (2004).

4 Kumar, S., Stecher, G. & Tamura, K. MEGA7: Molecular Evolutionary Genetics Analysis Version 7.0 for Bigger Datasets. *Molecular biology and evolution* **33**, 1870-1874, doi:10.1093/molbev/msw054 (2016).
